# Machine Learning for Diagnosis and Differentiation of Central Disorders of Hypersomnolence: A Systematic Review

**DOI:** 10.1111/ene.70661

**Published:** 2026-06-04

**Authors:** Annina Helmy, Livia G. Fregolente, Marc von Gernler, Rafael Morand, Julia van der Meer, Markus Schmidt, Stavroula Mougiakakou, Athina Tzovara, Claudio L. A. Bassetti

**Affiliations:** ^1^ Sleep‐Wake Epilepsy Center, NeuroTec, Department of Neurology Inselspital, Bern University Hospital, University of Bern Bern Switzerland; ^2^ Center for Experimental Neurology, Sleep Wake Epilepsy Center, NeuroTec, Department of Neurology Inselspital, Bern University Hospital, University of Bern Bern Switzerland; ^3^ Graduate School for Cellular and Biomedical Sciences University of Bern Bern Switzerland; ^4^ Graduate School for Health Sciences University of Bern Bern Switzerland; ^5^ Medical Library, University Library of Bern University of Bern Bern Switzerland; ^6^ ARTORG Center for Biomedical Engineering Research University of Bern Bern Switzerland; ^7^ Institute of Computer Science University of Bern Bern Switzerland; ^8^ Faculty of Medicine University of Bern Bern Switzerland

**Keywords:** central disorders of hypersomnolence, deep learning, idiopathic hypersomnia, machine learning, MSLT, narcolepsy, narcolepsy borderland, polysomnography, sleep EEG

## Abstract

**Background and Purpose:**

Central disorders of hypersomnolence (CDH) are, except for Narcolepsy Type 1 (NT1), difficult to diagnose and manage because of overlapping features and the lack of reliable biomarkers. Machine learning (ML) has the potential to improve diagnosis by detecting subtle physiological patterns and distinguishing between CDH subtypes. This review systematically explores current ML applications in CDH, assesses their limitations, and suggests future directions.

**Methods:**

Following PRISMA guidelines, MEDLINE, Embase, PsycINFO, IEEE Xplore, CINAHL, Web of Science, and Google Scholar (up to June 2025) were searched for studies using ML to classify or characterize CDH in adults. ML methods, data types, and diagnostic outcomes were extracted and analyzed.

**Results:**

Out of 3274 studies, 41 met the inclusion criteria (37 peer‐reviewed articles and 4 preprints). Data sources included neuroimaging (fMRI, PET), sleep assessments (MSLT, polysomnography), demographics, and standardized questionnaires. Supervised ML reliably identified known features, including early REM onset, hypocretin deficiency, and spectral EEG changes, showing strong performance for NT1 but limited generalizability across other CDH subtypes. Although many studies reported high accuracy, clinical relevance was often limited by rigid diagnostic labels that may not reflect the true complexity of CDH. Unsupervised learning uncovered heterogeneous phenotypes and exposed limitations in existing diagnostic labels.

**Conclusion:**

ML has the potential to improve CDH diagnosis. Deep learning models are promising for feature extraction; however, their black‐box nature and high data requirements hinder clinical application. Future advancements depend on large, diverse datasets, multimodal and longitudinal data, and close collaboration between clinicians and data scientists.

## Graphical abstract

This graphical abstract presents the review process (including 41 studies). It highlights the use of machine learning techniques in adults with CDH, such as unsupervised learning for phenotype discovery and supervised learning for diagnosis classification. The figure summarizes the strengths and limitations of each approach, along with key clinical and methodological challenges. Future directions should include longitudinal, multimodal datasets, enhancing diagnostic labels by adding follow‐up assessments, and promoting increased collaboration between clinicians and data scientists.

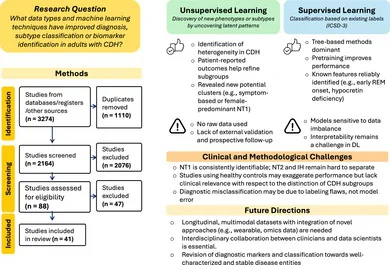

**Abbreviations**
CDH: Central Disorders of Hypersomnolence
CNN: Convolutional Neural Network
CSF: Cerebrospinal Fluid
DL: Deep Learning
EDS: Excessive Daytime Sleepiness
EEG: Electroencephalography
ESS: Epworth Sleepiness Scale
fMRI: functional Magnetic Resonance Imaging
GPT: Generative Pretrained Transformer
GRU: Gated Recurrent Unit
HC: Healthy Controls
HLA: Human Leukocyte Antigen
IH: Idiopathic Hypersomnia
ISS: Insufficient Sleep Syndrome
KLS: Kleine‐Levin Syndrome
LSTM: Long‐Short‐Term‐Memory Network
ML: Machine Learning
MLP: Multilayer Perceptron
MSLT: Multiple Sleep Latency Tests
NREM: Non‐Rapid Eye‐Movement
NT1: Narcolepsy Type 1
NT2: Narcolepsy Type 2
PET: positron emission tomography
PSG: Polysomnography
REM: Rapid Eye Movement
RF: Random Forest
RNN: Recurrent Neural Network
SVM: Support Vector Machine
XGBoost: Extreme Gradient Boosting

## 1 Introduction

One prominent group of sleep disorders, known as central disorders of hypersomnolence (CDH), is characterized by excessive daytime sleepiness (EDS) and/or excessive need for sleep (ENS) [1]. The International Classification of Sleep Disorders, 3rd edition (ICSD‐3), defines eight disorders: Narcolepsy Type 1 (NT1), Narcolepsy Type 2 (NT2), Idiopathic Hypersomnia (IH), Kleine‐Levin Syndrome (KLS), hypersomnia due to a psychiatric disorder, hypersomnia caused by a medical condition, hypersomnia resulting from medication or substances, and insufficient sleep syndrome (ISS) [2]. Despite distinct classifications, CDH frequently exhibits overlapping features, and current diagnostic tools have limited accuracy [3, 4, 5, 6].

Among CDH, NT1 is the most well‐characterized, thanks to biomarkers such as sleep‐onset rapid eye movement (REM) sleep periods (SOREMPs), positive HLA‐DQB1*06:02, and orexin deficiency in cerebrospinal fluid (CSF) [7]. NT1 also presents a distinct phenotype, including cataplexy, hallucinations, sleep paralysis, and disturbed nighttime sleep. In contrast, NT2 and IH lack reliable biomarkers and show significant clinical overlap, making diagnosis uncertain [7, 8, 9]. Longitudinal observations further underscore this uncertainty, showing that NT2 is often an unstable diagnosis over time and that some IH patients may even experience remission [10]. This diagnostic ambiguity, particularly among NT2, IH, and other non‐NT1 disorders, has led to the concept of the narcolepsy borderland (NBL), which includes the spectrum of poorly defined hypersomnolence conditions with overlapping symptoms and uncertain pathophysiology [7, 11].

The lack of specific and sensitive biomarkers, overlapping clinical features, and ongoing discussions around the limitations of the ICSD‐3 criteria for CDH highlight the need for more data‐driven diagnostic tools [1, 12, 13, 14]. Machine learning (ML) offers promising capabilities to address this gap by uncovering hidden patterns in complex datasets, thereby supporting biomarker discovery and diagnostic refinement. Deep learning (DL), a branch of ML, employs multi‐layered neural networks to extract features from raw data. The two main techniques within ML are unsupervised and supervised methods: unsupervised methods, such as clustering or dimensionality reduction, identify latent patterns in data not labeled with the classification of interest, while supervised methods use labeled data for prediction and classification tasks [15, 16, 17, 18]. ML has demonstrated increased usefulness in medicine for tasks like disease classification, clinical decision support, and image‐based diagnostics. These tools have the potential to enable earlier, more accurate diagnoses and more personalized treatment plans [19]. In the context of CDH, these capabilities could aid in more objective classification and help shape revision of diagnostic criteria. Although there is an increasing interest in ML techniques for sleep medicine, it remains unclear how both supervised and unsupervised approaches can best contribute to diagnosis, biomarker discovery, and subtype characterization of CDH.

To address this question, we performed a systematic review that aims to evaluate current evidence on the use of machine learning to improve diagnostic criteria and characterize clinical variability within CDH. Specifically, our work seeks to: (1) identify the data modalities used (e.g., clinical, electrophysiological); (2) describe the ML methods applied, assess their performance, and examine their role in biomarker discovery; and (3) analyze how these methods address diagnostic uncertainty, particularly within the NBL, highlighting key strengths and limitations.

## 2 Methods

### 2.1 Research Question

The research question of this review is: “What types of data and ML techniques have been applied to improve diagnostic accuracy, subtype classification, or biomarker identification in adults with central disorders of hypersomnolence?”

### 2.2 Inclusion and Exclusion Criteria

For the inclusion and exclusion criteria for this review, please refer to Table 1.

**TABLE 1:** Overview of inclusion and exclusion criteria.

Criteria	Inclusion	Exclusion
Population	humans, adults (> = 18 years) Diagnosed or suspected of having CDH according to ICSD‐3, including: NT1, NT2, IH, KLS, ISS.	Children, adolescents, or animal studies. Individuals with hypersomnolence secondary to psychiatric disorders, medical disorders, medications or substances.
Intervention/Exposure	Applications of ML or DL (e.g., supervised, unsupervised, reinforcement learning, neural networks, regression models when applied as predictive/diagnostic modeling with evaluation of performance such as AUC, sensitivity/specificity, cross‐validation). Aim must be related to developing innovative diagnostic tools, identifying biomarkers, clustering/classifying patient subtypes, or predicting diagnoses.	Studies that do not apply any ML/DL methods (including regression unless explicitly framed and evaluated as predictive/diagnostic modeling). Studies applying ML/DL for treatment, prognosis, or sleep staging without a direct connection to CDH diagnosis. Studies using statistical methods solely for association testing, group comparisons, or exploratory analysis without diagnostic aim or model performance reporting.
Comparator/Context	Any relevant clinical or research setting.	Studies set in irrelevant context (e.g., non‐sleep studies).
Outcome	Outcomes related to (diagnostic) performance. Identification of diagnostic features or clusters. Novel insights into heterogeneity or subtypes.	Studies where outcomes do not relate to diagnosis or classification.
Study Characteristics	Peer‐reviewed research studies. Preprints if they contain sufficient methodological detail. English language.	Reviews, editorials, letters, opinion pieces, or conference abstracts without full data. Non‐English language publications.
Other	Any ML/DL technique can be included (e.g., supervised, unsupervised, reinforcement learning, neural networks, SVMs, clustering etc.). Any modality can be included.	ML/DL which is applied to generic sleep features which are not contextualized within CDH.

### 2.3 Search Strategy

To identify all potentially relevant documents on the topic, comprehensive literature searches were designed and conducted across the following information sources:

- MEDLINE (Ovid) (Ovid MEDLINE(R) ALL (1946–04/06/2025))
- Embase (Ovid) (1974–04/06/2025)
- APA PsycInfo (Ovid) (1806–05/2025, Week 4)
- IEEE Xplore (IEEE)
- CINAHL (EBSCO) (CINAHL with Full Text (1963–Present))
- Web of Science Core Collection (Clarivate) (1900—Present)

An initial search strategy was developed in MEDLINE by a medical information specialist and tested against a list of core references to see if they were included in the search result. After refinement and consultation with the researchers, the information specialist developed complex search strategies for each source based on database‐specific controlled vocabulary (index terms/subject headings), when available, and free‐text terms, including synonyms, acronyms, and similar terms. In addition to the systematic search, Google Scholar was also searched. Four additional studies were identified by hand searching.

The following search concepts were applied: (1) “Hypersomnolence,” (2) “Machine Learning,” (3) “Diagnosis.” Studies involving animals, plants, and children were excluded using double‐negative search expressions in MEDLINE, Embase, and CINAHL. References related to specific diseases and treatments were excluded from MEDLINE and Embase through controlled vocabulary (Major Topics/Focus). The Google Scholar search and export of results were performed using Harzing's Publish or Perish 8 [20]. Although Google Scholar offers more than 11,000 records for our query, we exported the first 200 most relevant records, as we judged this to be sufficient for a supplementary search. All searches were run on 05 June 2025. The detailed final search strategies are available in the Supporting Information S3 and on searchRxiv.org [21, 22, 23, 24, 25, 26, 27].

### 2.4 Study Selection

**FIGURE 1:**
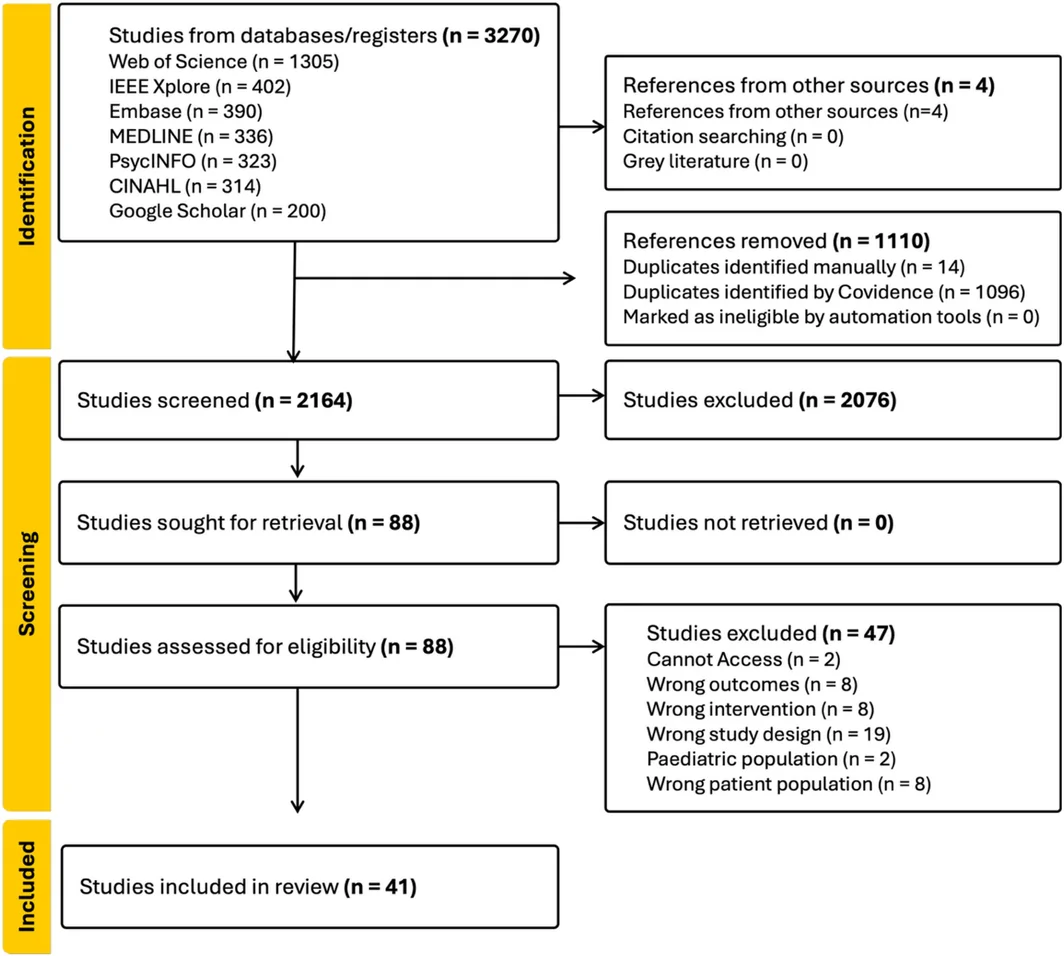
Overview of the selection process.

We exported all references identified by the search strategy from the databases. We imported them into Covidence, an online tool for managing systematic reviews, where duplicate records were removed before the screening. A team of up to four reviewers screened the articles. At least two reviewers reviewed each title and abstract independently to determine eligibility. Articles considered potentially relevant were screened in full text, with a minimum of two independent reviewers conducting the review. Any disagreements were resolved through discussion and consensus within the review team. The study selection process is summarized in the Preferred Reporting Items for Systematic Reviews and Meta‐Analysis (PRISMA) [28] flow diagram (Figure 1).

### 2.5 Data Extraction and Synthesis of Results

We extracted key variables from each study, including author(s) and publication year, study population (e.g., disorder type and sample size), clinical objective (e.g., diagnosis, classification, or biomarker identification), data type, preprocessing steps, learning type, machine learning approach/model, training and validation strategy, key results, and strengths and limitations reported by the authors. After extracting this data, we conducted a synthesis, grouping the studies by dataset, ML approaches, and study aim. This formed the basis for the narrative synthesis comparing supervised and unsupervised approaches across different data modalities and study objectives. Due to the heterogeneity of study designs, we informally evaluated methodological quality across four dimensions: validation strategy, sample size, class imbalance, and label reliability.

## 3 Results

### 3.1 Overview of Included Studies

A total of 3274 papers were found through the initial searches. After manual and automatic deduplication, as identified by Covidence, 2164 titles and abstracts were included for initial screening. Further filtering, conducted according to the defined inclusion and exclusion criteria, resulted in 88 studies for full‐text review. Following this screening, 47 studies were excluded (Figure 1). This resulted in a final inclusion of 41 studies (37 peer‐reviewed articles and four preprints). A detailed overview of these studies is provided in the Supporting Information S2.

### 3.2 Study Characteristics

**FIGURE 2:**
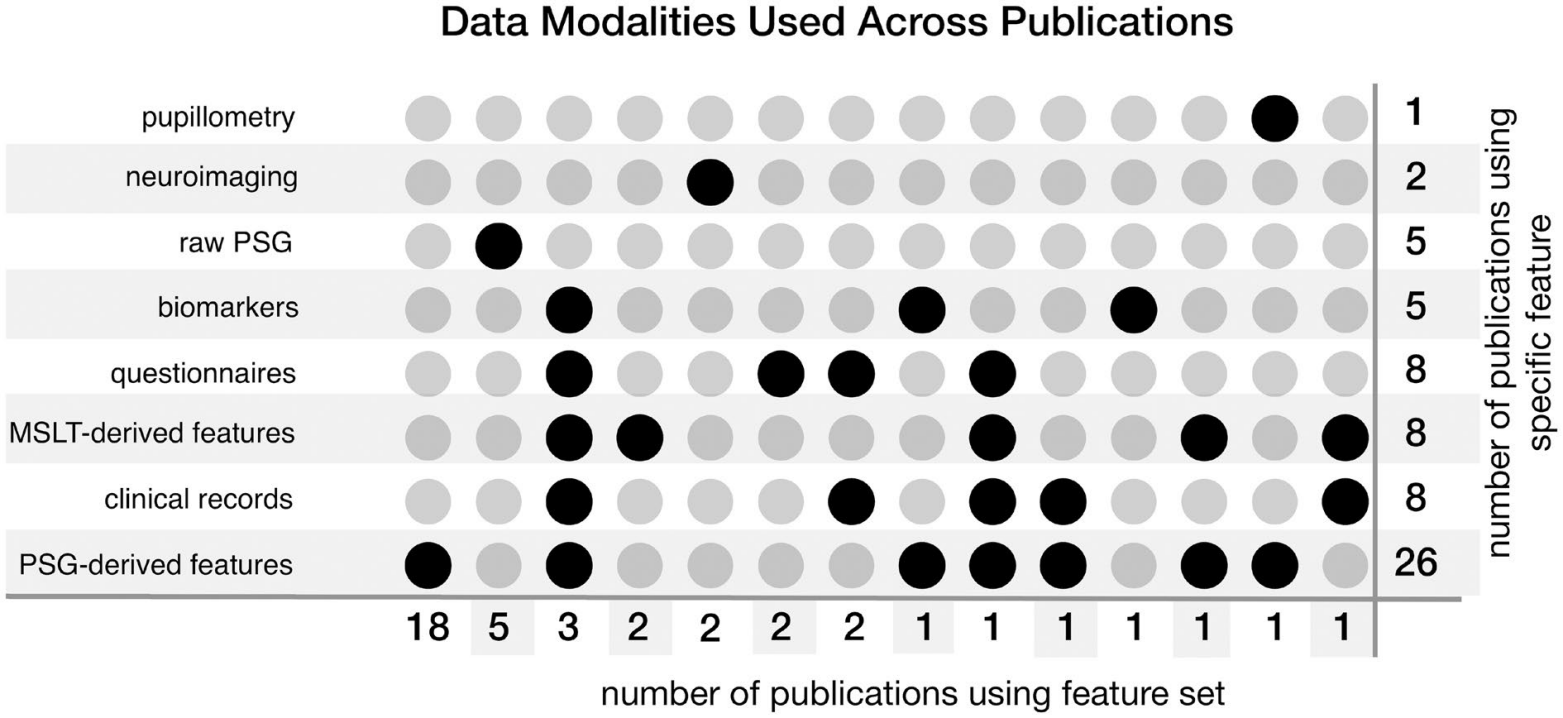
Displays combinations of data types (rows) used across various publications. Numbers on the bottom indicate the number of studies employing each specific modality combination. Numbers on the right show the number of publications in which a particular feature was used. Filled circles (black) indicate that the corresponding data type was used in that publication. The most common data types are PSG‐derived features and raw PSG, followed by clinical records and questionnaires. Single modalities are more frequently used, while combined modalities are less common. Biomarkers include HLA‐status, CSF‐hypocretin levels, proteomics, and neuroimaging (including PET and fMRI). PSG‐derived features consist of extracted power features from PSG channels, hypnograms, hypnodensity plots, statistical features, and more. Raw PSG refers to data input without extensive preprocessing.

Among the 41 studies, nine employed unsupervised and 32 supervised ML methods (Table 2, Table 3). Twelve studies used the Cyclic Alternating Pattern (CAP) dataset [29], which comprises polysomnography recordings from individuals with narcolepsy and other sleep disorders. The remaining studies used diverse data sources from private or open datasets. Figure 2 provides an overview.

**TABLE 2:** Overview of unsupervised machine learning methods used by the included studies.

Method	Application	Key characteristics	Strengths	Limitations
K‐Means	Identifying subgroups	Partitions data into k clusters by minimizing within‐cluster variance; each cluster represented by centroid (e.g., mean point, not necessarily a real observation)	Simple, efficient, works well when clusters are well‐separated	K must be predefined struggles with irregular cluster shapes
K‐Medoids	Identifying subgroups	Partitions data into k clusters using medoids (real observations) as centers; works with flexible distance metrics	More robust to outliers than k‐means; medoids are interpretable as real patients	K must be predefined may converge slower than K‐Means
Hierarchical Clustering	Identifying subgroups	Feature importance can be extracted	No need to predefine number of clusters	Computationally intensive on large datasets sensitive to noise in the data
Adaptive Resonance Theory (ART) Neural Network	Pattern recognition, clustering	Neural network that adaptively clusters inputs based on similarity, forms new clusters when no match found	Dynamic learning. If it learns something, it never forgets.	Sensitivity to vigilance parameters; less commonly used; interpretation can be complex.

**TABLE 3:** Overview of supervised machine learning methods used by the included studies. Information compiled from [15, 16, 17, 18].

Method		Key characteristics	Strengths	Limitations
Tree‐Based Methods		Use decision rules to split data into branches, forming a tree structure for classification or regression tasks.		
	Decision Trees	Splits data recursively using feature‐based rules	Interpretable, can handle mixed data types	Prone to overfitting, sensitive to small data changes
	Bagging (e.g., Random Forest)	Build multiple models on bootstrapped samples, aggregates results	Reduces overfitting; robust to noise	Slower to train, less interpretable
	Boosting (e.g., GBM, XGBoost, SGB)	Sequential ensemble of weak learners (typically trees) trained to minimize error.	High accuracy, handles missing data well	Sensitive to hyperparameters, computationally intensive
Instance‐Based Learning		Makes predictions by comparing new data points directly to stored training examples, no explicit model built.		
	K‐NN	Uses distance metric to find k closest labeled neighbors, use majority voting for classification	Easy to implement	Distance metric and choosing right k crucial, poor scalability with large datasets
Linear Models		Make predictions by assuming linear relationship between input feature and target vector.		
	Logistic Regression	Models probability of class membership via sigmoid function	Commonly used, interpretable coefficients	Less effective on non‐linear problems
	Linear Discriminant Analysis (LDA)	Projects data to maximize class separability		Assumes normality and equal covariance
Kernel‐based methods				
	Support Vector Machine (SVM)	Finds optimal boundary (hyperplane) between classes	Effective with high‐dimensional data	Harder to interpret; sensitive to tuning
Neural Networks		Computational models consisting of hidden layers of interconnected neurons that learn to map inputs to outputs by adjusting their internal parameters during training.		
	Multilayer Perceptron (MLP)	Fully connected layers	Simple architecture	Prone to overfitting on small datasets.
	Convolutional Neural Network (CNN)	Processes spatial/local patterns using convolutional filters	Effective for time series and image data. Requires fewer parameters than fully connected layers	Requires a lot of data.
	Recurrent Neural Network (RNN)	Retains information through hidden states over time	Suitable for sequential data	Struggles with very long sequences. Requires a lot of data.
	Long‐Short‐Term‐Memory Network (LSTM)	Designed for long‐term dependencies with memory cells and gates	Suitable for long sequential data. Better memory than RNN.	Complex to train, long training times. Still limited performance on very long sequences.
	Transformer	Self‐attention mechanism. Global context modeling.	Parallelizable. Captures long‐range dependencies.	High memory and computational costs for long sequences. Requires large training data.
	Generative Pretrained Transformer (GPT)	Transformer‐based model pre‐trained on massive text data. Can be efficiently fine‐tuned for Natural Language Processing tasks.	Strong transfer learning capabilities.	High computational cost. Performance depends on large‐scale data and fine‐tuning.

In terms of employed features for the ML algorithms, PSG‐derived features were the most common ones (*n* = 26 studies, Figure 2). These included hypnogram‐based features, sleep EEG power, hypnodensity plots, and more. This was followed by clinical records (*n* = 8), questionnaires (*n* = 8), and MSLT‐derived features (*n* = 8). Fewer studies employed raw PSG data (*n* = 5 studies), biomarkers (*n* = 5; e.g., HLA status and CSF levels), neuroimaging techniques (*n* = 2, Positron Emission Tomography (PET), Magnetic Resonance Imaging (fMRI)), or pupillometry (*n* = 1). Most studies relied on a single modality (*n* = 30, Figure 2, numbers on the bottom), with studies using only PSG‐derived features (*N* = 18) accounting for nearly half of all studies. Fewer studies combined multiple data sources (*n* = 11), highlighting limited use of multimodal data integration in CDH‐related ML research.

**FIGURE 3:**
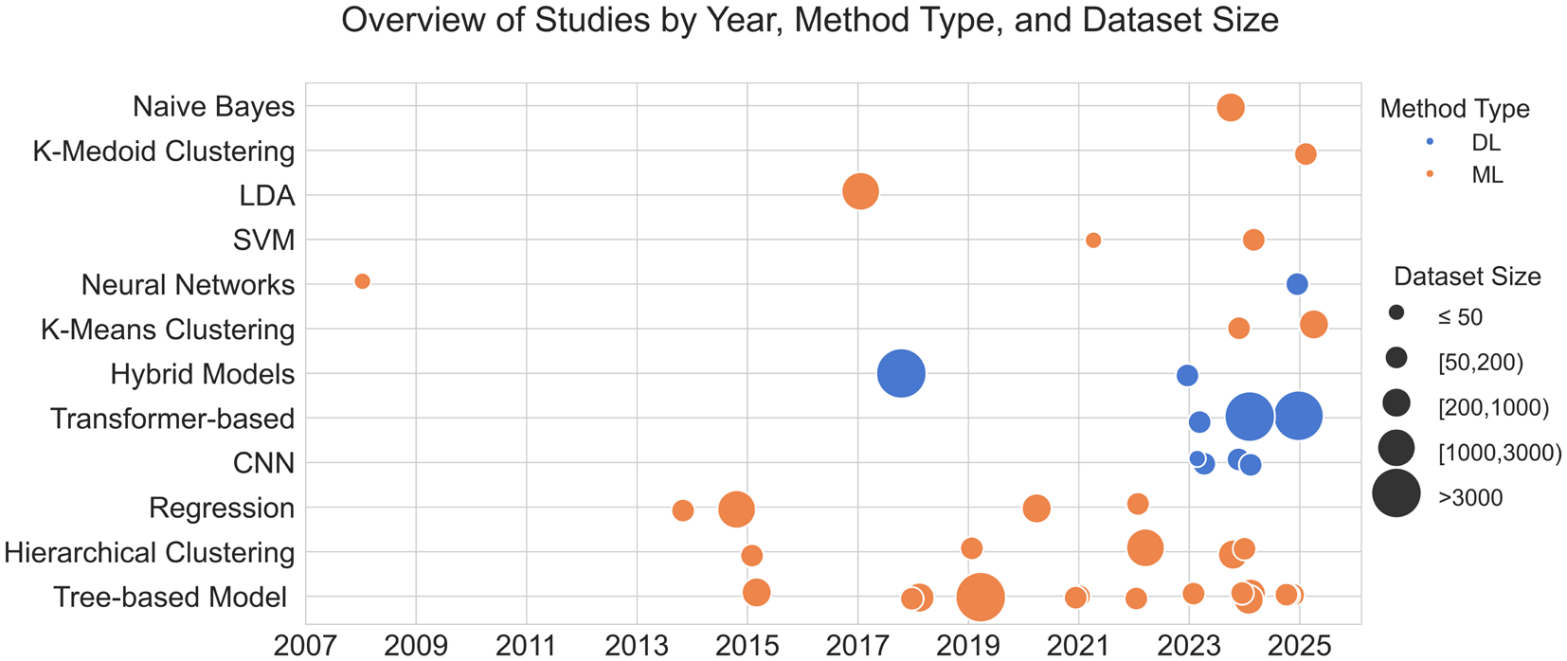
Overview of the included studies categorized by classifier type, dataset size, and publication year. Each point represents a study, placed according to its publication year on the x‐axis and algorithm group on the y‐axis. The color indicates whether the classifier is ML or DL, and the size of each scatter point reflects the dataset size used. Most DL studies emerged after 2020 and generally used larger datasets. Traditional machine learning (ML) methods were more common across different dataset sizes.

In terms of methodology, 33 studies used traditional ML, while eight studies employed more DL, typically requiring larger datasets. All but one DL study appeared in recent years, after 2021 (Figure 3).

### 3.3 Quality and Validation of Results in Included Studies

For unsupervised studies, cluster validity was assessed using a combination of internal metrics such as silhouette coefficient, elbow method, resampling procedures, and clinical judgment [30, 31, 32, 33]. Stability and consistency were evaluated through repeated model runs, randomization tests, and consensus clustering across subsamples [34, 35]. Two studies relied mainly on statistical analysis of input feature differences between observed clusters rather than formal clustering validation [36, 37]. One study additionally linked NT1 clusters to gray matter volumetric differences, providing external biological validation [30].

Regarding the validation strategy for the supervised studies, most relied on internal validation methods, using a single dataset and including k‐fold cross‐validation, usually 5‐ or 10‐fold, with some extending to repeated or stratified versions to reduce bias [38, 39, 40, 41, 42, 43, 44, 45, 46, 47, 48, 49, 50, 51, 52, 53, 54, 55]. Only five studies employed more rigorous methods, including external validation with independent datasets or cross‐center validation cohorts [56, 57, 58, 59, 60]. Three studies lacked sufficient validation detail for proper assessment, and one study did not report any validation procedures [61].

Studies using the CAP dataset (S1. CAP Dataset) showed less consistency in validation protocol. Several studies did not clarify whether subject‐level independence was maintained. Only three studies using the CAP dataset explicitly reported subject‐independent validation [62, 63].

### 3.4 Results of Unsupervised Machine Learning Applications

#### 3.4.1 Types of Unsupervised Methods Used

Among the included studies using unsupervised ML, three methods were applied (Table 2). Two studies used K‐Means clustering, which groups patients into a predetermined number of clusters as defined in advance [34, 35]. A more recent study employed k‐medoid clustering, which is similar to K‐Means but offers greater resistance to outliers [30]. Five other studies used hierarchical clustering, which does not require a predetermined number of clusters and instead relies on clinical interpretability to determine cutoff points [31, 32, 33, 36, 37]. One study used a less common method: the modified adaptive resonance theory neural network [64].

#### 3.4.2 Disorders and Datasets

Most studies using unsupervised learning methods aimed to identify subtypes within the CDH populations, though they varied in scope and composition. While most focused on NT1, NT2, and IH [30, 31, 32, 33, 34, 36, 37], one study extended beyond core CDH diagnoses to include ISS, hypersomnia due to a psychiatric disorder, or Excessive Daytime Sleepiness (EDS) not meeting CDH criteria [35], and one took a broader approach enrolling patients across hypersomnolence disorders without restriction to specific diagnoses [37]. Some studies further enriched their cohorts with healthy controls [32, 35], or reported comorbid Attention‐Deficit/Hyperactivity Disorder (ADHD) [32].

Diagnostic criteria and data availability also varied with one study applying ICSD‐2 classifications and lacking hypocretin measurements for NT1 [36]. Two studies also reported the level of diagnostic certainty, providing additional context for interpretation [31, 35]. Sample sizes ranged from over 1000 patients [31] to mid‐sized cohorts of 62–220 [30, 32, 33, 34, 35, 36, 37], with one study using a very small sample (*N* = 15) [64]. Most studies used multimodal datasets that combined objective sleep measures from PSG and MSLT with subjective data from questionnaires, along with demographic information, CSF hypocretin‐1 levels, and HLA typing [31, 34, 35]. Notably, two studies incorporated both objective and subjective measures [31, 34], and two other studies focused exclusively on subjective questionnaire metrics [35, 37]. One study focused on MSLT latency dynamics alone to reveal temporal patterns suggestive of IH subtypes [32].

#### 3.4.3 Main Findings

Unsupervised learning methods mainly adopt an exploratory approach, aiming to discover new subtypes and clarify feature differences among subgroups (Supporting Information S2, Table S2.1). All unsupervised ML studies identified patient clusters with shared characteristics. Among these, NT1 patients included in the populations were consistently recognized as a distinct phenotype, with two studies identifying an NT1 cluster primarily comprising female patients [31, 34]. A recent study further identified four NT1 subtypes based on age of symptom onset, weight gain, and symptom severity [30].

Clustering often revealed overlap between NT2 and IH. One study identified two groups of patients without cataplexy, consisting of patients with IH and NT2. One of these clusters showed higher HLA‐DQB1*0602 positivity and lower hypocretin‐1 levels, which the authors suggested may represent possible risk factors for disease progression towards NT1 [31]. This study also highlighted clinical differences, specifically in sleep drunkenness, subjective difficulty in awakening, and sleep need, suggesting that these features may help differentiate NT2 from IH [31]. Supporting these findings, another study found that NT2 and IH with short sleep duration tended to cluster together, while NT1 and IH with long sleep duration emerged as distinct when clustering on MSLT features, nap characteristics, and cataplexy symptoms [36].

Two studies using subjective measures reported clusters that reflect overall symptom severity rather than separate diagnostic categories [35, 37]. One distinguished a severely affected group with high fatigue, sleep drunkenness, and poor sleep quality, and a milder group with less functional impairment [35]. Another study similarly distinguished a more severe cluster with greater sleep inertia, depression, and functional impairment from a less severe phenotype [37]. The heterogeneity in IH was also examined. Clustering the MSLT nap dynamics identified two phenotypes: one characterized by generally longer sleep latencies, and another with longer morning sleep latencies that decreased in the afternoon [32]. Additional analyses suggested the presence of two nocturnal sleep phenotypes in individuals with IH: one characterized by deeper, more efficient sleep, and another by fragmented, less efficient sleep [33].

### 3.5 Results of Supervised ML Applications

#### 3.5.1 Types of Supervised Methods Used

A range of supervised ML methods have been applied across the included studies (Table 3). Tree‐based methods were the most common (*N* = 14 studies), including bagging approaches such as Random Forest and gradient boosting variants, and generally showed strong performance; however, they need more tuning and are computationally intensive [38, 39, 40, 41, 42, 43, 44, 45, 46, 47, 48, 56, 62, 65]. Five studies used linear models, such as regression [49, 57, 66, 67] and Linear Discriminant Analysis (LDA) [58]. These regression‐based approaches are highly interpretable but limited when linear assumptions are violated. Support Vector Machines (SVMs) were used in four studies and are well‐suited for high‐dimensional data; however, in two of these, SVMs served only as comparator models [39, 41, 62, 68].

Neural network‐based approaches, particularly Convolutional Neural Networks (CNNs), were applied to PSG and EEG data in four studies [50, 54, 61, 63]. Some studies integrated CNNs with Long Short‐Term Memory (LSTM) networks (*N* = 2) [51, 59] or Transformer architectures (*N* = 2) [52, 69] to capture additional temporal dependencies. In particular, multitask learning was applied in four studies by jointly learning model sleep staging and diagnosis, using advanced models such as CNN‐Transformers, Generative Pretrained Transformers (GPT), or hierarchical Transformer networks [52, 59, 60, 69].

#### 3.5.2 Disorders and Datasets

Studies varied widely in their patient populations and dataset sizes. The most common diagnostic groups across studies were NT1, NT2, and IH, often alongside healthy or non‐narcoleptic controls. Twenty‐six studies included healthy controls or non‐narcoleptic controls in their dataset [38, 39, 40, 41, 42, 45, 47, 50, 51, 52, 53, 55, 57, 58, 60, 61, 62, 63, 65, 67, 68, 69, 70]. Several studies also included conditions such as subjective EDS [48] and one focused exclusively on the Kleine‐Levin Syndrome [67]. Some studies used non‐CDH conditions as comparator groups, including OSA, insomnia, or broader undifferentiated sleep disorders. This variability in population and comparator selection makes it hard to directly compare model performance across studies.

Many studies performed binary classification within the CDH, for example, NT1 vs. NT2 (*N* = 5 studies) [44, 47, 48, 55, 56] or NT1 vs. IH (*N* = 4) [46, 47, 48, 56]. Sample sizes ranged from small cohorts (~30 individuals) to over 3000 patients (Figure 3) [46, 58]. Binary classification studies usually involved a variety of sample sizes (30 to over 700) [38, 43, 44, 45, 68], while multiclass studies drew from mid‐sized datasets covering multiple diagnoses [47, 49, 55, 65, 66]. Some studies leveraged large, multicenter datasets with thousands of recordings for pretraining [52, 59, 60]. Twelve studies have utilized the open‐source CAP dataset [29] (S1. CAP Dataset), which contains 108 overnight PSG recordings across various sleep disorders, including five cases of narcolepsy [39, 40, 41, 42, 50, 51, 53, 54, 61, 62, 63, 70]. Although this dataset was initially intended for CAP research, these studies applied it for disorder classification.

Regarding PSG input signals, seven studies used EEG only [39, 40, 41, 46, 51, 57, 61], while fourteen studies included combinations of EEG, EOG, EMG, ECG, nasal pressure, SpO2, or respiratory signals [42, 43, 45, 50, 52, 53, 54, 56, 58, 59, 62, 68, 69, 70]. One study relied solely on peripheral signals, excluding EEG entirely [63].

The majority of studies used PSG‐derived features, including spectral power, statistical measures, sleep architecture metrics, spindles, and slow waves [39, 40, 41, 43, 44, 45, 46, 47, 48, 53, 56, 57, 58, 61, 62, 65, 68]. Four studies incorporated hypnodensity features via automated sleep scoring algorithms [45, 47, 48, 56]. Two studies segmented overnight PSG data into quartiles, thereby incorporating time information into the feature set [47, 48]. In addition to physiological signals, some studies included questionnaire‐based features or symptom checklists [49, 60, 66]. A few studies did not use PSG, with two using neuroimaging (PET or fMRI) [38, 55], to detect brain activity patterns related to CDH, whereas one study analyzed the proteomic profiles [67].

Not all datasets included all the different sleep stages for classification. Overall, NREM3 and NREM4 were generally the most informative for multiclass classification [39, 40, 41], whereas NREM2 was identified as optimal for distinguishing between narcolepsy and controls [41]. One study using CAP phase B features reported the strong results for narcolepsy, highlighting the potential value of CAP‐specific markers [51].

#### 3.5.3 Main Findings

The supervised studies aimed to accomplish interconnected goals: classifying CDH subgroups, identifying key features and biomarkers, and gaining insights into subtype characteristics. Detailed performance metrics for each study are provided in Supplementary S2, Table S2.2. The subsequent sections highlight major findings, organized by diagnostic comparisons.

##### 3.5.3.1 Classification of NT1 vs. Healthy Controls

In terms of diagnostic performance, many studies consistently demonstrated high accuracy and specificity in differentiating NT1 from HC [38, 39, 42, 47, 51, 53, 54, 57, 58, 60, 61, 62, 63, 65, 69].

Regarding biomarker identification, two studies reported sleep EEG‐based feature differences between NT1 and HC, including REM‐wake dissociation, increased alpha power in REM, and reduced sigma and delta activity in Wakefulness and NREM1, respectively [57, 58]. Additionally, two studies also reported that REM sleep onset latency and sleep instability were the most discriminating [47, 65]. Resting‐state fMRI revealed consistent frontal and parietal changes in NT1, suggesting distributed neural alterations as potential imaging‐based biomarkers [38].

##### 3.5.3.2 Classification of NT1 vs. NT2

Studies differentiating NT1 from NT2 used physiological and imaging features. Two studies showed that REM‐related dynamics during MSLT, particularly the transitions from NREM2/NREM3 to REM sleep and the mixing of NREM1 and REM stages, can aid in subtype classification [56, 65], with one study improving classification accuracy by combining features from both the lights‐on and lights‐off periods of the MSLT [56]. One study showed that REM sleep onset tied with quarter‐night transitions in NREM2 and NREM3, was most important for classification [47].

Beyond classification performance, removing cataplexy and hypocretin levels from the feature set decreased model specificity, highlighting their diagnostic significance [44]. Additionally, weekend sleep duration emerged as a potential new noninvasive diagnostic feature, with NT1 patients showing shorter sleep duration on weekends than NT2 patients. An imaging study could classify NT1 vs. NT2 patients based on PET activity in the left basal ganglia, left Hesch's gyrus, and left striatum, consistent with previously reported metabolic differences between subtypes in these regions [55]., suggesting these regions as potential imaging biomarkers for subtype differentiation.

##### 3.5.3.3 Classification Within CDH

Questionnaire‐based models showed promise for screening purposes. One model detected multiple sleep disorders with high sensitivity but moderate specificity for ISS [49], and another identified narcolepsy risk with high specificity but lower sensitivity, using symptom checklists and daytime impact symptoms [66].

Six studies classified broader CDH subgroups such as NT2, IH, and subjective EDS, either in binary comparison against healthy controls or NT1, or in other subgroup comparisons [45, 46, 47, 48, 56, 68]. Two studies integrated the temporal information of overnight sleep dynamics using PSG features segmented into sleep quartiles. Both additionally integrated hypnodensity plot features, showing that ML‐derived features outperformed those from manual scoring [47, 48]. Two studies used sleep EEG spectral features to differentiate IH from NT1 [46, 47], while one study showed that NT2 and IH could be differentiated with moderate performance, highlighting subtle differences in sleep stability and structure [48].

Beyond classification performance, several studies provided insights into CDH subtype characteristics and potential biomarkers. One study highlighted that, compared to controls, IH was linked to increased sigma power in NREM2, more mixed wake/NREM1 epochs, and higher spindle and slow wave density, indicating changes in sleep microarchitecture [45]. Additionally, one study highlighted REM onset latency as the key feature classifying CDH groups, and sleep instability metrics were more informative in distinguishing NT1 from NT2 or controls [47].

## 4 Discussion

This systematic review examined the use of ML to improve diagnosis, classification, and biomarker discovery of CDH. Most studies included applied supervised learning methods to distinguish CDH subgroups; fewer explored unsupervised techniques for discovering latent phenotypes. Both approaches demonstrate considerable potential, yet also limitations, in advancing the classification of CDH beyond current clinical criteria.

### 4.1 Unsupervised Learning Studies: Strengths and Limitations

Unsupervised ML is a promising tool for uncovering phenotypic heterogeneity in CDH, especially for identifying subtypes that fall outside well‐defined diagnostic categories. Studies combined objective (i.e., EEG/PSG/imaging, etc.) and subjective (i.e., questionnaires) data, consistently identifying homogeneous NT1 clusters and revealing heterogeneity in NT2, IH, and other disorders within the NBL populations. Some clusters matched novel or unrecognized subtypes, such as predominantly female NT1 or symptom‐severity‐based NBL groups, suggesting gender differences in the clinical manifestation of NT1 and the potential influence of self‐perception of the disorder. The existence of a female NT1 subtype is particularly of interest, as it may reflect hormonal influences on disease manifestation. Patient‐reported measures (e.g., validated questionnaires assessing symptom severity or fatigue) offer valuable insights for distinguishing phenotypic subgroups and show that subjective patient reports can complement clinical measurements. Moreover, symptom severity can also reflect disease progression and, therefore, could also contribute to clinical assessment as a potential early marker for detection. However, several limitations remain. Most studies relied on features derived from hypnograms rather than raw data, which could overlook subtle physiological patterns and introduce bias, e.g., from manual scoring.

Additionally, clusters were typically evaluated through internal consistency or expert interpretation. Still, they lacked external validation (e.g., replication in an independent cohort) or prospective follow‐up (e.g., longitudinal tracking of outcomes), limiting their clinical applicability. Future research should connect data‐driven subtypes to long‐term outcomes to assess their clinical usefulness. Current unsupervised clustering studies indicated that novel (digital) biomarkers, such as more detailed physiological data, are needed to improve the characterization of CDH phenotypes.

### 4.2 Supervised Learning Studies: Assumptions and Feature Analysis

In supervised ML studies, established clinical and biological markers have consistently emerged as key predictors, including early REM onset, sleep stage dissociation, abnormal sleep stage transitions, and biomarkers such as cataplexy and hypocretin deficiency. While NT1 is the most distinct and readily classifiable group, performance drops for NT2 and IH, where overlapping features and diagnostic uncertainty make classification substantially more challenging. This performance gap, however, reflects the underlying uncertainty of the current disorder classification. NT1 is supported by robust, well‐defined biomarkers, making it more distinct. In contrast, NT2 and IH lack such markers, and their diagnostic categories remain uncertain.

Beyond classification performance, several studies contributed to biomarker identification. Spectral features, such as increased alpha activity during REM sleep, decreased sigma during wakefulness, and increased delta power during NREM1, were highlighted as particularly informative, especially in NT1. These findings indicate that ML can assist in confirming the diagnostic significance of known physiological markers, although their utility for NT2 and IH is still limited.

Supervised learning methods require datasets with definitive labels as ground truth. In the reviewed studies, these labels were typically based on the diagnostic criteria of the ICSD‐3. While necessary for training supervised models, this reliance on hard labels assumes the validity and stability of current diagnostic classifications. However, ongoing debates about the need for revised definitions and surrounding current diagnostic methods raise concerns about their use as ground truth [1, 12, 13]. As a result, supervised models might unintentionally reinforce a flawed framework and could also hinder the discovery of new biomarkers. This suggests that current ML models cannot yet be considered clinically ready for standalone diagnosis.

The disproportionate use of the CAP dataset [29] across 12 supervised studies warrants critical consideration. Although it is open source and therefore a valuable contribution to the limited number of open‐source datasets, it was primarily designed to illustrate cyclic alternating patterns across different pathologies, rather than to support disorder classification. It includes only 108 subjects across seven pathologies and additionally some healthy subjects, resulting in severe class imbalance and limiting the robustness of any model trained on it. Additionally, sleep staging follows the old Rechtschaffen and Kales [71] system rather than the current AASM [2] guidelines, with stages 3 and 4 not merged into NREM3. Being single‐center recordings from one sleep clinic, the dataset also lacks population diversity. These characteristics collectively limit the generalizability of findings derived from it to broader CDH populations.

Most supervised learning studies used tree‐based methods, which are inherently interpretable and make it easy to identify the most influential features. Importantly, many employed rigorous validation techniques, boosting the reliability of their results. However, a key concern remains that several studies reported performance metrics at the epoch level rather than at the more clinically relevant subject level. This can inflate overall accuracy but limits clinical relevance, as models may still misclassify individual patients when aggregating predictions. Additionally, supervised learning models are vulnerable to data bias, especially with imbalanced datasets [72]. In such datasets, accuracy can be misleading because models might just achieve high scores by favoring the majority class. A recommended practice is to report appropriate metrics of classification performance that can deal with imbalanced data, such as the F1 score, which accounts for class imbalance by combining precision and recall [72].

A common limitation across several studies was the choice of the comparator group. While many studies relied on healthy controls, others used non‐CDH conditions such as OSA, insomnia, or undifferentiated sleep disorders as comparators. In both cases, this provides limited clinical insight or utility on its own. The clinical interest and greater challenge lie in discriminating among CDH subtypes, especially those on the NBL, where overlapping symptoms and subtle physiological differences complicate diagnosis [7, 8, 9]. Classification against well‐separated or non‐CDH groups yields systematically higher accuracy than comparisons within the diagnostically ambiguous NBL spectrum. Reduced sensitivity in NBL subgroups likely reflects both the limited discriminative power of available features and the uncertainty inherent in current diagnostic labels, highlighting the importance of refining diagnostic labels, careful feature selection, and the overall complexity of CDH phenotyping. Taken together, the diversity of comparator groups and diagnostic entities across studies makes direct cross‐study performance comparisons unreliable, and reported performance metrics should be interpreted in this context.

### 4.3 Methodological Considerations and Challenges

A major challenge in applying ML to subgroup discovery is the limited availability of large, diverse, and well‐annotated datasets. Small datasets increase the risk of overfitting and decrease the generalizability of models. There is a need for easy access to large, high‐quality datasets that encompass diverse data modalities, populations, centers, and raw data recording setups, all while safeguarding patient privacy and data security. Within CDH, high‐quality dataset annotations are especially crucial because of the known diagnostic uncertainty [1, 12, 13]. The European Narcolepsy Network (EU‐NN) database provides a large sample of CDH‐specific multi‐modal data from many European sleep centers, but so far, it does not include raw PSG data [73]. Alternatively, PSG raw data unified from different cohorts lack in‐depth multi‐modal CDH‐specific clinical information [59]. The international Swiss Primary Hypersomnolence and Narcolepsy Cohort Study (iSPHYNCS) [74] aims to collect multi‐modal CDH‐specific clinical information from patients and healthy controls. Patients are followed up over 3 years, including biosamples, features from conventional sleep–wake studies, and longitudinal measures from consumer‐grade activity trackers, as well as omics‐based technologies. By re‐evaluating diagnoses along with the longitudinal measures, the study aims to improve label quality and identify robust biomarkers.

On the algorithmic side, DL has become increasingly prominent in research, offering advantages in feature extraction and classification. Hypnodensity plots, based on automatic sleep scoring, represent sleep stage probabilities rather than traditional sleep scoring with fixed labels, offering more detailed information than traditional sleep staging [75]. This probabilistic method captures important features like sleep‐stage dissociation, making it especially helpful for distinguishing NT1 [59]. Furthermore, a key trend has emerged, of pretraining models on related auxiliary tasks to improve their ability to generalize. Depending on how the auxiliary task is constructed, this can take the form of self‐supervised learning or transfer learning. Both approaches have been shown to improve EEG‐based classification tasks [76, 77]. An advanced example of transfer learning is SleepGPT, a transformer‐based model trained on hypnograms. By treating sleep staging as a sequence prediction task, SleepGPT detects macrostructural abnormalities like short REM latency or REM‐wake transitions without relying on raw signal data [60]. However, like most models, it relies on 30‐s epoch‐based annotations, which are historically rooted and may hide relevant microstructural dynamics.

These models remain, however, computationally intensive, and more importantly, their interpretability is limited. Some studies focus on highlighting model attention [59, 60], like attention maps or feature analysis, to improve feature interpretability such as emphasizing physiological markers for NT1, like short REM latency or abrupt REM transitions. Nevertheless, the “black‐box” nature of these models remains a concern. Integrating explainability methods such as attentional visualization or feature attribution, alongside clinician involvement in model development, will be essential.

Promising directions include the use of multimodal transformers and foundation models that can better handle complex and heterogeneous data. Semi‐supervised learning, which combines labeled and unlabeled data, also offers a practical bridge between supervised and unsupervised methods, making it well‐suited to the label‐ambiguous nature of CDH. Furthermore, using raw physiological signals instead of relying only on handcrafted or derived features could enable deep learning models to uncover hidden patterns and biomarkers that more traditional methods might overlook. Multimodal fusion remains underutilized but is vital for capturing the full complexity of CDH. Instead of focusing on features from isolated modalities (like PSG), future research should adopt a more holistic approach, examining how disorder dynamics evolve over time and across multi‐modal domains.

Alongside these technical advances, developing standardized evaluation frameworks, including external validation on independent cohorts and consistent performance reporting across demographic and clinical subgroups such as age and sex, is essential to ensure that new tools are accurate, fair, and safe.

Finally, while dataset sharing is often restricted by privacy constraints, making code and data openly available should become standard to enhance reproducibility.

### 4.4 Clinical Implications

Across studies, NT1 emerged as the most readily identifiable CDH subtype, consistent with its well‐defined clinical and biomarker profile. In contrast, NT2, IH, and other CDH subtypes remain diagnostically ambiguous. Many of the predictive features highlighted by ML models, such as REM onset latency or sleep‐stage dissociation, are already familiar to clinicians [7, 8]. However, ML‐based studies on these features show that they have an additional, algorithmic potential as significant discriminators, emphasizing their diagnostic value. Although current models are not yet suitable for standalone diagnosis, they show promise as clinical decision‐support tools. Unsupervised approaches extend this role further by serving as hypothesis‐generating tools that can uncover novel phenotypes and guide future biomarker research, thereby opening a complementary avenue for machine learning in clinical and research settings.

## 5 Conclusions

Our review emphasizes the need to integrate both objective (e.g., PSG, biomarkers) and subjective (e.g., questionnaires) assessments in CDH classification, while also highlighting the untapped potential of using temporal and long‐term wearable data. Moving forward, longitudinal, multimodal data collection paired with close collaboration between clinicians, data scientists, and ML experts will be essential.

With access to large‐scale, multimodal datasets, ML approaches hold strong potential to improve diagnostics and uncover clinically meaningful subtypes in CDH towards patient‐tailored treatment and management.

## Author Contributions

**Annina Helmy:** conceptualization, writing – original draft, methodology, writing – review and editing, formal analysis, investigation, visualization. **Julia van der Meer:** investigation, writing – review and editing. **Livia G. Fregolente:** writing – review and editing, investigation, conceptualization. **Markus Schmidt:** writing – review and editing. **Claudio L. A. Bassetti:** conceptualization, supervision, funding acquisition, writing – review and editing. **Marc von Gernler:** methodology, writing – review and editing, investigation. **Rafael Morand:** investigation, writing – review and editing. **Stavroula Mougiakakou:** writing – review and editing. **Athina Tzovara:** conceptualization, writing – review and editing, supervision, writing – original draft.

## Funding

This project is financially supported by two Swiss National Science Foundation project grants (320030_185362 and 32003B_215721).

## Conflicts of Interest

The authors declare no conflicts of interest.

## Supporting information


**Table S2:1** Results—Included studies using unsupervised approaches
**Table S2:2** Results—Included studies using supervised approaches.

## Data Availability

Data sharing not applicable to this article as no datasets were generated or analysed during the current study.
