# Supplementary material for: Machine Learning for Diagnosis and Differentiation of Central Disorders of Hypersomnolence: A Systematic Review
Source: Eur J Neurol. 2026 Jun 4;33(6):e70661. doi: 10.1111/ene.70661 (PMC13580826; doi:10.1111/ene.70661)
Supplement: Supplementary file 1 [file ENE-33-e70661-s001.docx]

**Supplementary Material** for “Machine Learning for Diagnosis and Differentiation of Central Disorders of Hypersomnolence: A Systematic Review”

*Annina Helmy^1,2,3,*^, Livia G. Fregolente^1,4,*^, Marc von Gernler^5^, Rafael Morand^1,2,3,6^, Julia van der Meer^1^, Markus Schmidt^1,2^, Stavroula Mougiakakou^6^, Athina Tzovara^2,7,+^ and Claudio L. A. Bassetti^8.1,+^*

Affiliations:

1. Sleep-Wake Epilepsy Center, NeuroTec, Department of Neurology, Inselspital, Bern University Hospital, University of Bern, Bern, Switzerland
2. Center for Experimental Neurology, Sleep Wake Epilepsy Center, NeuroTec, Department of Neurology, Inselspital, Bern University Hospital, University of Bern, Bern, Switzerland
3. Graduate School for Cellular and Biomedical Sciences, University of Bern, Bern, Switzerland.
4. Graduate School for Health Sciences, University of Bern, Bern, Switzerland.
5. Medical Library, University Library of Bern, University of Bern, Bern, Switzerland.
6. ARTORG Center for Biomedical Engineering Research, University of Bern, Bern, Switzerland
7. Institute of Computer Science, University of Bern, Bern, Switzerland
8. Faculty of Medicine, University of Bern, Bern, Switzerland

+ Co-senior authors, * Co-first author

Corresponding Author: Helmy Annina

Address: Sleep-Wake Epilepsy Center, Department of Neurology, Inselspital, University Hospital, Bern, Switzerland.

Email: [annina.helmy@students.unibe.ch](mailto:annina.helmy@students.unibe.ch)

Table of Contents

[S1. CAP Dataset	3](#_Toc230686686)

[S2. Results	4](#_Toc230686687)

[S3. Full Search Strategies	18](#_Toc230686688)

[Bibliography	30](#_Toc230686689)

## S1. CAP Dataset

The open-source CAP dataset was primarily recorded and annotated to create a publicly available database with carefully labeled Cyclic Alternating Pattern (CAP) examples across various pathophysiological contexts.^1^ The goal was to support the development of automated CAP analyzers and fundamental research on CAP dynamics. CAP is a recurring EEG pattern seen during NREM sleep, consisting of cyclic sequences where cerebral activation (phase A) is followed by periods of deactivation (phase B), separating two consecutive phase A periods.^2^ However, these twelve studies used the dataset for disorder classification.

The dataset includes 108 overnight polysomnography recordings collected at the Sleep Disorders Center of Ospedale Maggiore in Parma, Italy.^1^ It features 16 healthy individuals, 40 patients with Nocturnal Frontal Lobe Epilepsy (NFLE), 22 with REM behavior disorder (RBD), 10 with Periodic Leg Movements (PLM), 9 with insomnia, five with narcolepsy, four with Sleep-disordered Breathing (SDB), and 2 with bruxism. Sleep stages were annotated using the older Rechtschaffen and Kales system (stages 0-5)^3^ . Notably, stages 3 and 4 were not merged into NREM3 as per current AASM guidelines.^4^

## S2. Results

*Table S2.1.: Results – Included studies using unsupervised approaches*

| Unsupervised Approaches | | | | | | |
| --- | --- | --- | --- | --- | --- | --- |
| Author, Year | Aim | Classifier | | Dataset | Input | Key Findings |
|  |  | Type | Algo. |  |  |  |
| Gool et al. (2022)^5^ | Identify data-driven clusters in CDH | ML | Hierarchical Clustering | N = 1078  unmedicated adolescents ≥13 year,  Patients with NT1, NT2, IH | *multimodal*  97 variables incl. PSG, MSLT, clinical symptoms, demographics, CSF-hypocretin-1, HLA, subjective measures | *7 clusters*  4 with cataplexy (2 marked by sleep drunkenness, difficulty awakening, sleep attacks); 1 female-dominant NT1 cluster; 2 without cataplexy—one showing high HLA positivity and low hypocretin. |
| Morand et al. (2025)^6^ | Identify phenotypic variability within CDH using questionnaires and metadata | ML | K-Means Clustering | N = 220  NT1 51, NBL 136, HC 33 | *multimodal*  ten different clinical questionnaires and demographic variables for clustering | *4 Clusters*  1 healthy, 1 typical NT1, 1 mild NLB, and 1 severe NBL cluster. Psychiatric symptoms and poor sleep hygiene were associated with greater severity in the NBL. |
| Aellen et al. (2024)^7^ | Data-driven approach for identification of SWDs | ML | K-means clustering | N = 141  78 NT1, 19 NT2, 44 IH  *This study tested also other cohorts not discussed here.* | *multimodal*  ~300 variables incl. clinical records, PSG, MSLT, actigraphy | CDH cohort: 4 clusters with 2 related to NT1, one primarily IH, one mixture NT2 and IH. 1 NT1 clusters dominated by females. |
| Maruo et al. (2024)^8^ | Identify central hypersomnia and ADH using MSLT features and clustering of temporal patterns | ML | Hierarchical clustering | N = 200  NT1: 17, NT2: 25,  97 IH, subjective EDS, not objectively proven: 61.  Within included cohort 93 with ADH | *Unimodal*  MSLT sleep latencies (in seconds) across 4 naps per subject | Nine clusters were identified: NT1/NT2 mainly in one. Temporal patterns improve diagnostic separation. IH and NT2 aren't well-separated, though IH shows a wider range of sleep latency than NT2. |
| Liu et al. (2008)^9^ | Classify OSA and narcolepsy using pupil size and EEG theta via NN | ML | Modified ART2 NN | N = 15  5 OSA, 4 narcolepsy, 6 controls | *Multimodal*  EEG theta activity + pupil diameter (2-sec window); 11.5 min recording in a dark room with task of staying awake | 91% accuracy (OSA vs. controls); 90% (narcolepsy vs. controls); theta-pupil coupling reflects alertness differences |
| Sonka et al. (2015)^10^ | Test the ICSD-2 classification of NT1, NT2, IH with/without long sleep time | ML | Hierarchical clustering | N = 96 25 IHw/oLST, 26 IHwLST, 23 NT1, 22 NT2  *(ICSD-2; LST = Long Sleep Time)* | *Multimodal*  Unwanted nap count/duration, irresistible unwanted naps, difficulty waking from naps, cataplexy, MSLT mean latency, MSLT number of SOREMPs | 3 clusters:  (1) IH without LST and NT2, (2) IH with LST, (3) NT1  NT1 and IH with LST are distinct.  Supports merging NT2 and IH without LST. |
| Mengmeng et al. (2025)^11^ |  | ML | K-Medoids | N = 158 NT1 (drug-naïve, HLA-DQB1*06:02+) | *Multimodal*  52 variables: demographcy, NT1 clinical manifestations, PSG and MSLT features, genetic phenotypes, questionnaire scores | 4 NT1 phenotypes:  (1) pre-teen onset, mild subjective EDS but severe objective sleepiness, (2) teen onset + large weight gain, (3) teen onset + less weight gain, (4) adult onset with highest ESS, more OSA comorbidity |
| Cook et al. (2019)^12^ |  | ML | Hierarchical Clustering | N = 62 with DSM-5 Hypersomnolence Disorder (narcolepsy, OSA, circadian disorders excluded) | *Monomodal*  Self-reported measures (Self-report total sleep time, ESS score, SIQ score). | 2 clusters: (1) more severe; greater EDS, sleep inertia, depression, worse function, worse vigilance, longer PSG sleep; (2) less severe; lower subjective symptoms.  Hopkins = 0.46 (marginal clusterability). |
| Baier et al. (2024)^13^ |  |  | Hierarchical Clustering | N = 69 IH patients | *Monomodal*  PSG features such as time spent in REM, NT1, N2, N3 sleep, and Sleep Efficiency | 2 clusters: (1) increased N3, sleep efficiency, decreased N2: better sleep continuity, less WASO, higher prevalence of one SOREMP; (2) less N3, more N2, less SE: lower sleep maintenance.  IH may encompass ≥2 nocturnal sleep phenotypes: “increased sleep pressure” vs “qualitative sleep disturbance”. |

Table S2.2. Results – Included studies using supervised approaches.

| Supervised Approaches | | | | | | | | | | | | | |
| --- | --- | --- | --- | --- | --- | --- | --- | --- | --- | --- | --- | --- | --- |
| Author, Year | Aim | Classifier | | | | N | Diagnostic Groups | | Input Modality | Performance | Key Findings | | Notes |
|  |  | Type | | | Algo. |  |  |  |  |  |  |  |  |
| Sharma et al. (2021)^14^ | Classification of sleep disorders using EEG | ML | | | EBoost, EBT, SVM, KNN  (binary and multiclass) | 77 | Subset CAP  Insomnia, NT1, NFLE, PLM, RBD, SDB, HC | | Unimodal  EEG (central, frontal): wavelet decomposition (Triplet Half-Band Filter); Hjorth parameters from 8 subbands | *Multiclass:* overall 91.3% accuracy (N3, EBoost); narcolepsy class: 97.65% accuracy, recall = 0.73  *Narcolepsy vs. Healthy:* 98.21% accuracy, AUC = 1.0, k = 0.96 | N3 stage most informative for multiclass classification; combined EEG channels outperformed single channel for NT1 vs Healthy. | | Epoch-based results; CAP dataset; class imbalance noted, |
| Dimitriadis et al. (2021)^15^ | Classification of sleep disorders using cross-frequency coupling (CFC) features | ML | | | Random Forest | 106 | Subset CAP  Insomnia, NT1, NFLE; PLM, SDB, Bruxism, HC | | Unimodal  EEG (occipital, P4–O2); CFC features (PAC and AAC) extracted across sleep stages | *Multiclass*: 74% accuracy  *Narcolepsy*: Precision: 100%, Recall: 20%, F1-score: 33.3% | Sleep Stage 4 (NREM4) was the most informative stage, using Non-CAP epochs. | | Results are subject-level, using AASM2 guidelines for sleep staging. |
| Babu et al. (2024)^16^ | Sleep disorder classification using EEG using FFT | DL | | | Xception CNN | 108 | CAP  Insomnia, NT1, NFLE, PLM, RBD, SDB, HC, Bruxism | | Unimodal  Using FFT to convert EEG (frontal and central) into frequency spectrogram | Overall accuracy: 98.62%  *Narcolepsy:* Precision 0.93, Recall 0.78, F1 score 0.85, 98.06% accuracy | - | | Results are epoch-based. |
| Wadichar et al. (2023)^17^ | Hierarchical DL model for 1) healthy vs unhealthy, 2) sleep disorder identification | DL | | | 1D-CNN +LSTM | 108 | CAP  Insomnia, NT1, NFLE; PLM, SDB, Bruxism, HC | | Unimodal  EEG (central) 2-second segments. Only NREM sleep; CAP phase-specific | *Multiclass classification:*  - test accuracy (holdout): 90.55%  - test accuracy (10-fold CV): 83.05 ± 1.19%  *Healthy vs. Unhealthy:*  - test accuracy (holdout): 91.45%  - test accuracy (10-fold CV): 83.94±0.99%  *Narcolepsy* (F1-score, holdout): 90.33% (holdout) | CAP phase B most informative for narcolepsy detection (93.31 % accuracy). Both CAP phases for multiclass classification. | | Results are epoch-based. |
| Newaz et al. (2025)^18^ | Sleep disorder classification using time/frequency features | ML + DL | | | MLP, Random Forest, XGBoost, SVM (binary and multiclass) | 108 | CAP  Insomnia, NT1, NFLE, PLM, RBD, SDB, HC, Bruxism | | Unimodal  EEG (central)  Time-domain, frequency-domain, complexity, non-linear features. | *Multiclass*: 86% accuracy, F1 = 0.86 best with NREM4 and MLP.  *Narcolepsy vs. Healthy*: 94% accuracy, F1 = 0.88 | NREM4 most informative for multiclass classification, using MLP. NREM2 optimal for NT1 vs. HC discrimination. | | Results probably epoch-based. |
| Jain et al. (2024)^19^ | Sleep disorder classification using EEG and EOG | ML | | | SVM using radial basis function | 108 | CAP  Insomnia, NT1, NFLE, PLM, RBD, SDB, HC, Bruxism | | *Multimodal*  EEG (occipital) or EOG.  Features: spectral, statistical, nonlinear (entropy, Higuchi fractal dimension), wavelet-based, spindle count, poincaré features | *Narcolepsy vs. Healthy:*  EEG: 100%  EOG: 50% | Gridded distribution entropy most discriminative for NT1 vs. HC across all sleep stages; EEG substantially outperforms EOG for narcolepsy classification | | Subject-level results; Non-CAP epochs; only binary NT1 vs. HC reported for ancrolepsy. |
| Jain et al. (2025)^20^ | Classify multiple sleep disorders and assess sleep stagings role using only EEG + EOG | ML | | | LEE  combined Light-GBM-EOG-EEG (gradient boosting decision tree using EOG and EEG) with disambiguation using binary classifiers | 95 | Subset CAP dataset  Insomnia, NT1, NFLE, PLM, RBD, SDB, HC | | *Multimodal*  EEG (occipital) and EOG.  Features: ZRC (EEG), MMD (EOG), entropy, Poincaré, statistical & non-linear features | *7-class classification*  *LightGBM-EEG:* Slow-Wave sleep + Wake sleep 78.5% acc. *LightGBM*- *EOG:* 83% acc. (sleep-stage indep.).  *LEE classifier:* 93.3% acc.  Post-processing refines NT1 vs. SDB: 94.4% acc. | Combined EEG+ EOG (Lee classifier) outperforms either modality alone; REM/NREM3 ratio thresholding as post-processing step improves NT1 vs. SDB discrimination*.* | Results are subject-based.  EOG outperforms EEG alone in this multiclass setting. Post-processing adds clinically meaningful refinement. | |
| Sharma et al. (2022)^21^ | Classification of sleep disorders | ML | | | Ensemble Bagged Tree (EBT) | 66 | Subset CAP dataset  Insomnia, NT1, NFLE, PLM, RBD, HC | | *Multimodal*  EOG, EMG  wavelet decomposition using biorthogonal filter bank. Hjorth parameters. | *Multiclass* : 94.3% acc. with N3, 87.5% acc. for sleep-independent data using EMG and EOG  *Narcolepsy vs. Healthy:* EOG + EMG features (N3 sleep): acc. 97.6%, Cohen’s kappa of 0.952. Precision: 0.964, recall: 0.956, F1: 0.979. | N3 most informative for both multiclass and binary classification. | | *Results are epoch-based.* |
| Albathan (2024)^22^ | Sleep disorder classification with EEG and EOG | ML + DL | | | Bidirectional GRU (feature extractor) with Ensemble Bagged Tree Classifier | 108 | CAP  Insomnia, NT1, NFLE, PLM, RBD, SDB, HC, Bruxism | | *Multimodal*  EEG and EOG converted into spectrograms | *Narcolepsy*: 97.6% acc., and 0.97 AUC  Overall accuracy: 96.59%, AUC 0.966 (10-fold CV). |  | | *Results are epoch-based.* |
| Cheng et al. (2023)^23^ | Simultaneous sleep staging and disorder detecting | DL | | | VGG16 CNN + shallow neural network | 108 | CAP  Insomnia, NT1, NFLE, PLM, RBD, SDB, HC, Bruxism | | Multimodal  EEG, EOG, EMG.  STFT used to create spectrograms, used as inputs. | *Overall accuracy:* 99.01% when using simultaneous training for disorder detection.  Single modality: ECG with 93.74% acc.  Trained on fusion of modalities:  98.93% acc. | Fusion of modalities and labels improved performance over single-modality methods. | | *Results are epoch-based.* |
| Singh et al. (2023)^24^ | Classification of sleep disorders using EMG, EOG, ECG. | DL | | | Light 1D CNN | 44 | Subset CAP  Insomnia, NT1, PLM, SDB, HC | | Multimodal  EOG, EMG, ECG. Sleep-stage independent. | *Narcolepsy vs. HC*: F1 score: 0.83, AUC: 0.83, recall: 1.0 | Best if all signals combined. | | *Results are subject-level.* |
| Wang et al. (2024)^25^ | Sleep disorder classification using multi-modal PSG signals | DL | | | MAF-CNN (Multimodal Attention-Fusion Convolutional Neural netowr) | 108 | CAP  Insomnia, NT1, NFLE; PLM, HC | | Multimodal  EEG, EOG, ECG, EMG | *Multiclass classification:* Accuracy: 99.56%, F1: 99.49%, Kappa: 0.9942. EEG+ECG+EMG: 99.59%.  All disorder classes >99% accuracy. |  | | *Results are epoch-based.* |
| Olsen et al. (2017)^26^ | Sleep stage dissociation in PSG as a diagnostic marker for NT1 | | ML | LDA | | *> 2500* | *Multiple datasets*  250 NT1, >1000 controls | *Multimodal*  EEG, EOG, EMG  38 features from power bands, power band ratios, Hjorth parameters | | *Binary classification*: Up to 95% specificity; sensitivity of 94% in one, 38% in another dataset. | 2D sleep state space interpretable marker  NT1 shows sleep stage dissociation, especially in REM and wake states. | |  |
| Christensen et al. (2015)^27^ | Characterize EEG across sleep stages in narcolepsy vs. controls | | ML | Lasso-regularized logistic regression | | *1373* | *Multiple datasets*  NT1: 155, Non-Narcoleptic Patients: 1218 | *Unimodal*  63 EEG power spectral features | | NT1 vs. Non-NT1:  Accuracy 70.3%, AUC 48.0%, sensitivity 26.3%, and specificity 71.5% | 13 important features identified. NT1: increase alpha in REM, decrease sigma in wake, decrease delta in N1 | |  |
| Andersen et al. (2025)^28^ | Identify NT1 markers using features from MSLT “lights-on” and “lights-off” periods | | ML | Random Forest | | *177* | 69 NT1, 37 NT2, 56 IH, 25 sH  across 3 centers | *Multimodal*  Standard sleep metrics, hypodensity, microsleep, and stage mixing features during lights-on and lights-off periods. | | *Classification*: NT1 vs. sIH: 92% specificity (lights-on)  NT1 vs. NT2: specificity 0.95 (combined features) | NT1 shows shorter microsleep latency and more N1–REM mixing compared to other diagnostic groups. Combining lights-on and lights-off periods improved diagnostic performance. | |  |
| Liu et al. (2015)^29^ | Identification of REM sleep stage transition patterns as biomarkers for narcolepsy | | ML | Decision Tree | | 259 | 35 NT1, 39 NT2, 26 relatives, 159 non-narcoleptic controls | *Multimodal*  PSG/MSLT features (REM transitions, SOREMPs, sleep latency) | | *Classification*: sensitivity of 94.3% and specificity 82.4% for NT1; 82.1% sensitivity and 83% specificity for NT2.  REM from non-N2/N3 in MSLT high sensitivity for NT1 and NT2 (94.3%, 82.1%), but low specificity (61.6%). | Altered REM sleep transitions from non-N2/N3 stages as novel biomarker for both NT1 and NT2; high sensitivity but limited specificity when used as standalone features. | | *Mutliclass (NT1, NT2, relatives, non-narcoleptic controls). Subject-level results.* |
| Chin et al. (2024)^30^ | Classify narcolepsy subtypes using PET imaging | | ML | Naïve Bayes + Term Variance (for feature selection) | | 314 | 204 NT1, 90 NT2, 20 NT1 + schizophrenia, 26 HC | *Unimodal*  Positron emission tomography (PET) data  18F-FDG PET brain ROIs | | >99% acc. with 3 ROIs | (left basal ganglia, Heschl’s gyrus, striatum); high performance with minimal features. | |  |
| Fulong et al. (2018)^31^ | Identify fALFF-based brain activity markers for NT1 | | ML | Classification and Regression Tree | | 111 | 51 NT1: 26 adults and 25 juveniles; 60 controls) | *Unimodal*  Resting-state fMRI (fALFF from ROIs) | | Adults: AUC 0.99, acc. 94%; | key ROIs: decreased values in bilateral medial superior frontal gyrus, bilateral inferior parietal lobule, supra-marginal gyrus; increased bilaterial sensorimotor cortex and middle temporal gyrus activity may reflect compensatory processes. | |  |
| Gaultney et al. (2014)^32^ | Improving self-report survey accuracy for narcolepsy risk via symptom checklist + ESS | | ML | Binary logistic regression | | 172 | 45 narcolepsy, 19 narcolepsy and insomnia, 3 narcolepsy and OSA, 1 with all disorders, 104 with insomnia and/or OSA | *Multimodal*  Symptom checklist based on Sleep-50 + Epworth Sleepiness Scale scores (ESS) + daytime impact symptoms | | Specificity ≥96% using symptoms and daytime impact. | Adding ESS improved classification accuracy, sensitivity for narcolepsy. | |  |
| Pan et al. (2024)^33^ | Predict comorbid NT1 in OSA patients with EDS | | ML | GBM | | 246 | NT1: 82 OSA: 164 | *Multimodal*  PSG + clinical features (11 selected via LASSO) | | *Classification:* GBM AUC = 0.88, with sensitivity 0.76, specificity 0.83 for NT1 in patients with OSA | *Top features*: age of onset, limb movement index, sleep latency, NREM2%, OSA severity | |  |
| Zhang et al. (2018)^34^ | Identify features differentiating NT1 vs. NT2 | | ML | SGB | | 702 | 104 NT2, 598 NT1  *Statistical analyses performed on subsets due to missing features.* | *Multimodal*  317 clinical features incl. PSG, MSLT, CSF hypocretin, HLA, questionnaires | | *Classifier*: acc. 0.9943, specificity 1, sensitivity 0.993 (with cataplexy features); 0.6 balanced accuracy, sensitivity 0.98, specificity 0.23 (removing cataplexy) | *Top predictors*: cataplexy, hypocretin levels, REM latency (MSLT), HLA, sleep efficiency. *Novel*: weekend sleep length. | |  |
| Son et al. (2021)^35^ | Identify NT2 biomarkers via physiological network connectivity during REM | | ML | SVM | | 30 | 15 NT2, 16 HC | *Multimodal*  EEG, EOG, EMG, ECG, RESP  36 signal pairs; Time Delay Stability (TDS) scores | | SVM accuracy 93% binary classification. | Stable REM connectivity in NT2: especially $\theta$ -heartrate, heartrate-eye, chin-respiratory | |  |
| Coz et al. (2025)^36^ | Identify EEG markers of IH using EEG features | | ML | XGBoost | | 128 | 80 IH (with long sleep time), 48 controls | *Multimodal* Hypnodensity metrics, spectral features, infralow fluctuations, sleep spindle and slow wave characteristics | | *Classification:* AUC 74% (balanced dataset), accuracy-IH: 64,% accuracy-control 69% | IH shows increased sigma power in NREM2, more mixed wake/NREM1 sleep epochs, more sleep spindles, higher slow wave density | |  |
| Buettner et al. (2019)^37^ | Differentiate Narcolepsy vs. IH using 10-min EEG | | ML | Random Forest | | 3168 | 10-min EEGs 1584 narcolepsy with hypocretin deficiency, 1584 IH | *Unimodal*  EEG power in 99 bands (0.5-50 Hz, with 0.5 Hz step); 6 channels | | *Classification (10-fold CV)*: Accuracy 74.87% with sensitivity 76.26% and specificity 73.48%, kappa 49.75%. | Most predictive are bands up to 3 Hz (delta), and 14.5-15 Hz (beta). 0.5-2.5 Hz increased in NT1, 14-14.5 Hz decreased in NT1 compared to IH | |  |
| Vilela et al. (2024)^38^ | Identify time-resolved PSG features distinguish NT1 from NT2, IH, and controls | | ML | Random Forest  (binary classification) | | 350 | drug-free individuals:  114 NT1, 90 NT2, 105 IH, 41 controls | *Multimodal*  Whole/quarter-night power band features, sleep metrics, sleep stage transitions, hypnodensity | | *Classification*: combined feature sets yield AUC 0.89-0.91 for NT1 vs. other | *Most discriminative features:* REM sleep onset latency, sleep instability for NT1 vs. NT2/controls, spectral features for NT1 vs. IH.  time-resolved metrics improved performance | |  |
| Cesari et al. (2023)^39^ | Differentiate central hypersomnolence disorders using manual + AI derived PSG features | | ML | Random Forest  (binary classification) | | 143 | 40 NT1, 26 NT2, 23 IH, 54 sEDS | *Multimodal*  1003 manual + AI-derived PSG features from manual hypnograms, hypnodensity (YASA), transitions, bouts, stability and fragmentation of sleep stages, and distribution across quartiles | | *Classification*: NT1 vs. NT2 with 0.74±0.04 acc., 0.79±0.05 F1-score. Higher average performances for NT1 vs. IH/sEDS (0.89±0.09, 0.93±0.06 acc.). NT1 vs rest with an acc. 0.88±0.04, and F1 score of 0.80±0.04. IH vs. sEDS with acc. 0.57±0.11, but low sensitivity 0.3±0.25. | AI-derived features outperformed manual scoring in classification.  Confirms sleep instability in patients with NT1. No precise biomarker of NT2 or IH could be identified. | |  |
| Hédou et al. (2022)^40^ | Identify CSF and serum proteomic bioamrkers for Kleine-Levin Syndrome (KLS) | | ML | Lasso-based | | 164 (CSF), 91 (Serum) | With CSF:  30 KLS, 134 controls  With Serum:  26 KLS, 65 controls | *Multimodal*  Proteomic profiles (28 CSF, 141 serum proteins) | | AUC 0.90 (CSF), 1.0 (serum); | strong diagnostic potential with novel biomarker identification (serum + CSF) | |  |
| Schwartz et al. (2020)^41^ | Develop/validate digital sleep questionnaire sleep disorder identification | | ML | ElasticNet (regularized logistic regression) | | 247 | 60 Insomnia, 53 DSPS, 43 ISS, 17 None, 7 other | *Multimodal*  15-200 self-reported survey items and demographics features | | *Model performance ISS (5-fold CV)*: sensitivity 82.2±7.5%, specificity 63.6±7.5%. Overall acc. 69.6±4.7% | *Predictive features*: high likelihood of dozing, early morning/nocturnal awakenings, unrefreshed feeling in the morning, difficulty awakening on weekdays. | |  |
| Deng et al. (2025)^42^ | Large-scale generalizable PSG model for automated sleep staging and diagnosis of neuropsychiatric disorders | | DL | CNN + Transformer | | *24’000* | *16 datasets*  N = 24’000 PSG recordings  Fine-tuned on HANG7 dataset  N = 84 individuals (33 HC, 51 narcolepsy) | Multimodal  EEG, EOG, EMG | | *Classification:*  88% accuracy for narcolepsy detection on subject-level | Robust cross-center performance | |  |
| Yu et al. (2024)^43^ | Develop a SleepGPT for sleep staging and sleep disorder diagnosis | DL | | | Self-supervised GPT (SleepGPT) for pretrianing + supervised Hierarchical Transformer Network (HTN) for classification | *~8000* | *9 datasets*  Pretrained on ~8000 individuals from multiple datasets  Tested on dataset with 82 NT1, 43 other hypersomnia, 282 non-narcolepsy control | | *Unimodal*  Hypnograms | *Classification:* NT1 vs. others (using HTN): balanced acc. 92.81%, sensitivity 91.46% specificity 94.15%, | Interpretable attention highlights pathological patterns | |  |
| Stephansen et al.(2018)^44^ | Automated sleep staging and development of PSG-based biomarker for NT1 | DL | | | Ensemble of CNN + LSTM models for feature extraction; Gaussian Process Classifier for classification | *~3000* | *10 datasets*  N = ~3000 PSGs  For classification: NT1 vs. multiple different disorders from different datasets | | Multimodal  EEG, EOG, EMG (preprocessed signals as input) for pretraining. Hypnodensity features, clinically relevant features for NT1 classification. | *Classification:*  sensitivity of 91-93%, specificity of 91-96%. | Adding HLA, sensitivity achieved 90%, specificity increased to 99%. | |  |
| Wang et al. (2023)^45^ | Automatic narcolepsy diagnosis using PSG with sleep staging as auxiliary task | DL | | | CNN + Transformer (multitask deep learning) | 77 | 50 narcolepsy, 27 controls | | Multimodal  EEG, EOG, ECG, EMG, nasal pressure | *Classification:*  using EEG and EOG results in acc. of 79.71% with an F1-score of 84.47%  EEG alone resulted in highest F1-score (85.45%), with 78.94% accuracy. | sleep staging as auxiliary task improved performance | | *Performance metrics based on 10 min recordings.* |

## **S3. Full Search Strategies**

Database: Ovid MEDLINE(R) ALL (1946 – 04/06/2025)

Search date: 05/06/2025

Search Strategy:

----------------------------------------------------------------------

ID	Search	Hits

-----------------------------

1	"disorders of excessive somnolence"/ or idiopathic hypersomnia/ or kleine-levin syndrome/ or narcolepsy/ or cataplexy/	9033

2	((excessive or increased or daytime or day-time or diurnal) adj3 (sleepiness or drowsiness or somnolence)).ti,kf.	2984

3	(cataleptic attack* or cataplex* or disrupted nighttime sleep or disrupted nocturnal sleep or Gelineau* or Henneberg or insufficient nocturnal sleep or insufficient sleep syndrome or Kleine Levin or narcolep* or neurolepsy or paroxysmal sleep or pseudonarcolep* or sleep inertia? or sleeping beauty syndrome or sleep epilepsy).ti,ab,kf.	7231

4	((hypersomn* or hyper-somn* or hypersleepiness) adj3 (idiopath* or primary)).ti,ab,kf.	565

5	or/1-4 [Hypersomnolence]	13160

6	Artificial Intelligence/ or Machine Learning/ or Deep Learning/ or Clustering Algorithms/ or Machine Learning Algorithms/	131078

7	(machine learning or artificial intelligence or deep learning).ti,ab,kf.	262674

8	exp Supervised Machine Learning/ or exp classification algorithms/ or decision trees/ or random forest/	27515

9	(supervised learning or semisupervised learning or "learning from labeled data" or linear regression or logistic regression or support vector machine or SVM or decision tree? or random forest? or k-nearest neighbor or KNN or gradient boosting or Extreme Gradient Boost? or XGBoost or AdaBoost or Light Gradient Boosting Machine or LightGBM).ti,ab,kf.	722274

10	Representation Machine Learning/	0

11	(semi-supervised learning or self-supervised learning or representation learning or contrastive learning).ti,ab,kf.	6809

12	exp Unsupervised Machine Learning/	1327

13	(unsupervised learning or kmeans or k-means or hierarchical clustering or agglomerative clustering or DBSCAN or autoencoder).ti,ab,kf.	27405

14	Reinforcement Machine Learning/ or Transfer Machine Learning/	39

15	(reinforcement learning or few-shot learning or transfer learning or foundational models).ti,ab,kf.	17453

16	Neural Networks, Computer/	62072

17	(deep neural network or convolutional neural network or CNN or recurrent neural network or RNN or long short-term memory or LSTM or attention mechanism or BERT or GPT or graph neural network or GNN).ti,ab,kf.	66922

18	exp Ensemble Learning/	151

19	(ensemble learning or multimodal learning or ensemble model aggregation or iterative model building or categorical boosting? or CatBoost).ti,ab,kf.	4722

20	exp Dimensionality Reduction/	31573

21	(Dimensionality Reduction or Singular Value Decomposition or Linear Discriminant Analys#s or principal component analysis or PCA or "uniform manifold approximation and projection" or UMAP or t-distributed stochastic neighbor embedding or t-SNE or manifold learning or non-negative matrix factori#ation or NMF).ti,ab,kf.	122696

22	((digital or data-driven) adj3 ph?enotyping).ti,ab,kf.	771

23	or/6-22 [Machine Learning]	1161105

24	diagnosis/ or diagnosis, computer-assisted/ or diagnosis, differential/ or "diagnostic techniques and procedures"/ or clinical laboratory techniques/ or "Sensitivity and Specificity"/	896672

25	(distinguish* or diagnos* or determin* or differentiat* or discriminat* or recogni* or classif* or cluster* or predict* or screen* or detect* or identif* or ph?enotyp*).ti,ab,kf.	15517431

26	diagnos*.fx.	4450224

27	or/24-26 [Diagnosis]	17424917

28	and/5,23,27 [DOES and AI and Diagnosis]	555

29	exp *Parkinson Disease/ or exp *Alzheimer Disease/ or exp *Dementia/ or exp *Depression/ or exp *Schizophrenia/ or exp *Sleep Apnea Syndromes/ or exp *Obesity/ or exp *Heart Failure/ or exp *Neoplasms/ or exp *Autism Spectrum Disorder/ or exp *Insomnia/ or Modafinil/	4523303

30	(Birth Cohort/ or exp Child/ or exp Infant/) not (exp adult/ or Adolescent/)	1530647

31	(exp Animals/ or exp Animal Experimentation/ or exp Animal Structures/ or exp models, animal/ or exp Plants/ or exp Plant Structures/ or exp Fungi/) not exp humans/	5964622

32	or/29-31	11585717

33	28 not 32 [exclusion of certain populations]	336

----------------------------------------------------------------------

***************************

Database: Ovid Embase (1974 -- 04/06/2025)

Search date: 05/06/2025

Search Strategy:

----------------------------------------------------------------------

ID	Search	Hits

-----------------------------

1	disorders of excessive somnolence/ or hypersomnia/ or idiopathic hypersomnia/ or exp hypersomnolence/ or exp narcolepsy/ or cataplexy/	19391

2	((excessive or increased or daytime or day-time or diurnal) adj3 (sleepiness or drowsiness or somnolence)).ti,kf.	5177

3	(cataleptic attack* or cataplex* or disrupted nighttime sleep or disrupted nocturnal sleep or Gelineau* or Henneberg or insufficient nocturnal sleep or insufficient sleep syndrome or Kleine Levin or narcolep* or neurolepsy or paroxysmal sleep or pseudonarcolep* or sleep inertia? or sleeping beauty syndrome or sleep epilepsy).ti,ab,kf.	11550

4	((hypersomn* or hyper-somn* or hypersleepiness) adj3 (idiopath* or primary)).ti,ab,kf.	1156

5	or/1-4 [Hypersomnolence]	24840

6	machine learning/ or artificial intelligence/ or exp classification algorithm/ or classifier/ or supervised machine learning/ or exp support vector machine/ or k nearest neighbor/ or decision tree/ or random forest/ or logistic regression analysis/ or linear regression analysis/	583518

7	(machine learning or artificial intelligence or deep learning).ti,ab,kf.	301041

8	(supervised learning or semisupervised learning or "learning from labeled data" or linear regression or logistic regression or support vector machine or SVM or decision tree? or random forest? or k-nearest neighbor or KNN or gradient boosting or Extreme Gradient Boost? or XGBoost or AdaBoost or Light Gradient Boosting Machine or LightGBM).ti,ab,kf.	1018321

9	semi supervised machine learning/ or exp "feature learning (machine learning)"/ or exp deep learning/ or contrastive learning/	84547

10	(semi-supervised learning or self-supervised learning or representation learning or contrastive learning).ti,ab,kf.	7637

11	unsupervised machine learning/	4154

12	clustering algorithm/ or hierarchical clustering/ or k means clustering/ or agglomerative hierarchical clustering/	21536

13	(unsupervised learning or kmeans or k-means or hierarchical clustering or agglomerative clustering or DBSCAN or autoencoder).ti,ab,kf.	36771

14	learning algorithm/ or exp "reinforcement learning (machine learning)"/ or "transfer learning (machine learning)"/	28007

15	(reinforcement learning or few-shot learning or transfer learning or foundational models).ti,ab,kf.	19036

16	artificial neural network/ or exp autoencoder/ or deep neural network/ or convolutional neural network/ or graph neural network/ or recurrent neural network/ or long short term memory network/ or attention mechanism/ or generative pretrained transformer/	120950

17	(deep neural network or convolutional neural network or CNN or recurrent neural network or RNN or long short-term memory or LSTM or attention mechanism or BERT or GPT or graph neural network or GNN).ti,ab,kf.	76823

18	ensemble learning/ or boosting machine learning algorithm/	525

19	(ensemble learning or multimodal learning or ensemble model aggregation or iterative model building or categorical boosting? or CatBoost).ti,ab,kf.	5199

20	exp dimensionality reduction/ or principal component analysis/ or "uniform manifold approximation and projection"/ or t distributed stochastic neighbor embedding/ or manifold learning/ or locally linear embedding/ or non-negative matrix factorization/	107988

21	(Dimensionality Reduction or Singular Value Decomposition or Linear Discriminant Analys#s or principal component analysis or PCA or "uniform manifold approximation and projection" or UMAP or t-distributed stochastic neighbor embedding or t-SNE or manifold learning or non-negative matrix factori#ation or NMF).ti,ab,kf.	164078

22	((digital or data-driven) adj3 ph?enotyping).ti,ab,kf.	854

23	or/6-22 [Machine Learning]	1656564

24	exp diagnosis/ or diagnostic accuracy/ or exp diagnostic value/ or computer assisted diagnosis/ or diagnostic test accuracy study/ or diagnostic test/ or "sensitivity and specificity"/	9361851

25	(distinguish* or diagnos* or determin* or differentiat* or discriminat* or recogni* or classif* or cluster* or predict* or screen* or detect* or identif* or ph?enotyp*).ti,ab,kf.	20031450

26	diagnos*.fx.	3843527

27	or/24-26 [Diagnosis]	24215279

28	and/5,23,27 [DOES and AI and Diagnosis]	1216

29	*Parkinson disease/ or *Alzheimer disease/ or exp *dementia/ or exp *depression/ or exp *schizophrenia/ or exp *sleep apnea syndromes/ or exp *obesity/ or exp *heart failure/ or exp *neoplasm/ or exp *autism/ or exp *insomnia/ or modafinil/	6384899

30	(birth cohort/ or embryo/ or fetus/ or exp juvenile/) not (adult/ or institutionalized adult/ or aged/ or aged hospital patient/ or frail elderly/ or institutionalized elderly/ or very elderly/ or middle aged/ or young adult/)	3169670

31	(book or chapter or conference or conference abstract or conference paper or "conference review" or editorial or letter or note).pt.	9565424

32	or/29-31	16353568

33	28 not 32 [exclusion of certain publication types and populations]	402

34	limit 33 to "humans only (removes records about animals)"	390

----------------------------------------------------------------------

***************************

Database: Ovid APA PsycInfo (1806 -- 05/2025, Week 4)

Search date: 05/06/2025

Search Strategy:

----------------------------------------------------------------------

ID	Search	Hits

-----------------------------

1	hypersomnia/ or kleine levin syndrome/ or narcolepsy/ or catalepsy/	2761

2	((excessive or increased or daytime or day-time or diurnal) adj3 (sleepiness or drowsiness or somnolence)).tw.	5092

3	(cataleptic attack* or cataplex* or disrupted nighttime sleep or disrupted nocturnal sleep or Gelineau* or Henneberg or insufficient nocturnal sleep or insufficient sleep syndrome or Kleine Levin or narcolep* or neurolepsy or paroxysmal sleep or pseudonarcolep* or sleep inertia? or sleeping beauty syndrome or sleep epilepsy).tw.	3316

4	((hypersomn* or hyper-somn* or hypersleepiness) adj3 (idiopath* or primary)).tw.	258

5	or/1-4 [Hypersomnolence]	8346

6	artificial intelligence/ or exp machine learning/ or exp machine learning algorithms/	45381

7	(machine learning or artificial intelligence or deep learning or supervised learning or semisupervised learning or "learning from labeled data" or linear regression or logistic regression or support vector machine or SVM or decision tree? or random forest? or k-nearest neighbor or KNN or gradient boosting or Extreme Gradient Boost? or XGBoost or AdaBoost or Light Gradient Boosting Machine or LightGBM or semi-supervised learning or self-supervised learning or representation learning or contrastive learning or unsupervised learning or kmeans or k-means or hierarchical clustering or agglomerative clustering or DBSCAN or autoencoder? or reinforcement learning or few-shot learning or transfer learning or foundational models or deep neural network or convolutional neural network or CNN or recurrent neural network or RNN or long short-term memory or LSTM or attention mechanism or BERT or GPT or graph neural network or GNN or ensemble learning or multimodal learning or ensemble model aggregation or iterative model building or categorical boosting? or CatBoost or Dimensionality Reduction or Singular Value Decomposition or Linear Discriminant Analys#s or principal component analysis or PCA or "uniform manifold approximation and projection" or UMAP or t-distributed stochastic neighbor embedding or t-SNE or manifold learning or non-negative matrix factori#ation or NMF or ((digital or data-driven) adj3 ph?enotyping)).tw.	163694

8	or/6-7 [Machine Learning]	178027

9	exp Diagnosis/ or exp Computer Assisted Diagnosis/	265190

10	(distinguish* or diagnos* or determin* or differentiat* or discriminat* or recogni* or classif* or cluster* or predict* or screen* or detect* or identif* or ph?enotyp*).tw.	2476180

11	or/9-10 [Diagnosis]	2549286

12	and/5,8,11 [DOES and AI and Diagnosis]	323

----------------------------------------------------------------------

***************************

Database: IEEE Xplore

Search date: 05/06/2025

Search results: 402

Search Strategy:

----------------------------------------------------------------------

ID	Search	Hits

-----------------------------

("Full Text & Metadata":"excessive somnolence" OR "Full Text & Metadata":hypersomnolence OR "Full Text & Metadata":"idiopathic hypersomnnia" OR "Full Text & Metadata":"narcolep*" OR "Full Text & Metadata":cataplexy OR "Full Text & Metadata":cataleptic OR "Full Text & Metadata":hypersleepiness OR "Full Text & Metadata":neurolepsy OR "Full Text & Metadata":"paroxysmal sleep" OR "Full Text & Metadata":"sleep inertia*" OR "Full Text & Metadata":"sleeping beauty syndrome" OR "Full Text & Metadata":"sleep epilepsy")

AND

("Full Text & Metadata":"machine learning" OR "Full Text & Metadata":"artificial intelligence" OR "Full Text & Metadata":"deep learning" OR "Full Text & Metadata":"supervised learning" OR "Full Text & Metadata":"semisupervised learning" OR "Full Text & Metadata":"linear regression" OR "Full Text & Metadata":"logistic regression" OR "Full Text & Metadata":"support vector machine" OR "Full Text & Metadata":SVM OR "Full Text & Metadata":"decision tree" OR "Full Text & Metadata":"random forest" OR "Full Text & Metadata":"k-nearest neighbor" OR "Full Text & Metadata":KNN OR "Full Text & Metadata":gradient boost* OR "Full Text & Metadata":XGBoost OR "Full Text & Metadata":AdaBoost OR "Full Text & Metadata":LightGBM OR "Full Text & Metadata":"semi-supervised learning" OR "Full Text & Metadata":"self-supervised learning" OR "Full Text & Metadata":"representation learning" OR "Full Text & Metadata":"contrastive learning" OR "Full Text & Metadata":"unsupervised learning" OR "Full Text & Metadata":kmeans OR "Full Text & Metadata":"k-means" OR "Full Text & Metadata":"hierarchical clustering" OR "Full Text & Metadata":"agglomerative clustering" OR "Full Text & Metadata":DBSCAN OR "Full Text & Metadata":autoencoder OR "Full Text & Metadata":autoencoders OR "Full Text & Metadata":"reinforcement learning" OR "Full Text & Metadata":"few-shot learning" OR "Full Text & Metadata":"transfer learning" OR "Full Text & Metadata":"foundational models" OR "Full Text & Metadata":"deep neural network" OR "Full Text & Metadata":"convolutional neural network" OR "Full Text & Metadata":CNN OR "Full Text & Metadata":"recurrent neural network" OR "Full Text & Metadata":RNN OR "Full Text & Metadata":"long short-term memory" OR "Full Text & Metadata":LSTM OR "Full Text & Metadata":"attention mechanism" OR "Full Text & Metadata":BERT OR "Full Text & Metadata":GPT OR "Full Text & Metadata":"graph neural network" OR "Full Text & Metadata":GNN OR "Full Text & Metadata":"ensemble learning" OR "Full Text & Metadata":"multimodal learning" OR "Full Text & Metadata":"ensemble model aggregation" OR "Full Text & Metadata":"iterative model building" OR "Full Text & Metadata":"categorical boosting" OR "Full Text & Metadata":CatBoost OR "Full Text & Metadata":"Dimensionality Reduction" OR "Full Text & Metadata":"Singular Value Decomposition" OR "Full Text & Metadata":"Linear Discriminant Analys?s" OR "Full Text & Metadata":"principal component analys?s" OR "Full Text & Metadata":PCA OR "Full Text & Metadata":"uniform manifold approximation and projection" OR "Full Text & Metadata":UMAP OR "Full Text & Metadata":"t-distributed stochastic neighbor embedding" OR "Full Text & Metadata":"t-SNE" OR "Full Text & Metadata":"manifold learning" OR "Full Text & Metadata":"non-negative matrix factori?ation" OR "Full Text & Metadata":NMF OR "Full Text & Metadata":"digital phenotyping" OR "Full Text & Metadata":"data-driven phenotyping")

----------------------------------------------------------------------

***************************

Database: CINAHL with Full Text (1963 -- Present)

Search date: 05/06/2025

Interface: EBSCOhost Research Databases

Expanders: Apply equivalent subjects

Search Modes: Find all my search terms

Search Screen: Advanced Search

Search Strategy:

----------------------------------------------------------------------

ID	Search	Hits

-----------------------------

S1	(MH "Disorders of Excessive Somnolence+")	2475

S2	XB ((excessive OR increased OR daytime OR day-time OR diurnal) N3 (sleepiness OR drowsiness OR somnolence))	3653

S3	XB ("cataleptic attack*" OR cataplex* OR "disrupted nighttime sleep" OR "disrupted nocturnal sleep" OR Gelineau* OR Henneberg OR "insufficient nocturnal sleep" OR "insufficient sleep syndrome" OR "Kleine Levin" OR narcolep* OR neurolepsy OR "paroxysmal sleep" OR pseudonarcolep* OR "sleep inertia#" OR "sleeping beauty syndrome" OR "sleep epilepsy")	1713

S4	XB ((hypersomn* OR hyper-somn* OR hypersleepiness) N3 (idiopath* OR primary))	144

S5	S1 OR S2 OR S3 OR S4	5956

S6	(MH "Artificial Intelligence") OR (MH "Artificial Intelligence, Generative") OR (MH "Machine Learning+") OR (MH "Neural Networks (Computer)+") OR (MH "Decision Making, Computer Assisted+") OR (MH "Algorithms+")	116267

S7	XB ("machine learning" OR "artificial intelligence" OR "deep learning" OR "supervised learning" OR "semisupervised learning" OR "learning from labeled data" OR "linear regression" OR "logistic regression" OR "support vector machine" OR SVM OR "decision tree#" OR "random forest#" OR "k-nearest neighbor" OR KNN OR "gradient boosting" OR "Extreme Gradient Boost#" OR XGBoost OR AdaBoost OR "Light Gradient Boosting Machine" OR LightGBM OR "semi-supervised learning" OR "self-supervised learning" OR "representation learning" OR "contrastive learning" OR "unsupervised learning" OR kmeans OR "k-means" OR "hierarchical clustering" OR "agglomerative clustering" OR DBSCAN OR autoencoder# OR "reinforcement learning" OR "few-shot learning" OR "transfer learning" OR "foundational models" OR "deep neural network" OR "convolutional neural network" OR CNN OR "recurrent neural network" OR RNN OR "long short-term memory" OR LSTM OR "attention mechanism" OR BERT OR GPT OR "graph neural network" OR GNN OR "ensemble learning" OR "multimodal learning" OR "ensemble model aggregation" OR "iterative model building" OR "categorical boosting#" OR CatBoost OR "Dimensionality Reduction" OR "Singular Value Decomposition" OR "Linear Discriminant Analysis" OR "principal component analysis" OR PCA OR "uniform manifold approximation and projection" OR UMAP OR "t-distributed stochastic neighbor embedding" OR t-SNE OR "manifold learning" OR "non-negative matrix factorization" OR NMF OR ((digital OR data-driven) N3 ph#enotyping))	251518

S8	S6 OR S7	344062

S9	S5 AND S8	396

S10	(MH "Diagnosis+")	2283610

S11	XB (distinguish* OR diagnos* OR determin* OR differentiat* OR discriminat* OR recogni* OR classif* OR cluster* OR predict* OR screen* OR detect* OR identif* OR ph#enotyp*)	2578179

S12	S10 OR S11	3788049

S13	S5 AND S8 AND S12	335

S14	(MH "Animals+") NOT (MH "Human")	91336

S15	(MH "Child+" OR MH "Minors (Legal)" ) NOT (MH "Adult+" OR MH "Adolescence+" OR MH "Childbearing Age")	450920

S16	S13 NOT (S14 OR S15)	314

----------------------------------------------------------------------

***************************

Database: Web of Science Core Collection

Entitlements:

- WOS.SCI: 1900 to 2025

- WOS.AHCI: 1975 to 2025

- WOS.ESCI: 2020 to 2025

- WOS.ISTP: 1990 to 2025

- WOS.SSCI: 1900 to 2025

- WOS.ISSHP: 1990 to 2025

Search date: 05/06/2025

Search Strategy:

----------------------------------------------------------------------

ID	Search	Hits

-----------------------------

#1 TI=(distinguish* OR diagnos* OR determin* OR differentiat* OR discriminat* OR recogni* OR classif* OR cluster* OR predict* OR screen* OR detect* OR identif* OR ph$enotyp*) OR AB=(distinguish* OR diagnos* OR determin* OR differentiat* OR discriminat* OR recogni* OR classif* OR cluster* OR predict* OR screen* OR detect* OR identif* OR ph$enotyp*)	Results: 26237944

#2 TS=("machine learning" OR "artificial intelligence" OR "deep learning" OR "supervised learning" OR "semisupervised learning" OR "learning from labeled data" OR "linear regression" OR "logistic regression" OR "support vector machine" OR SVM OR "decision tree$" OR "random forest$" OR "k-nearest neighbor" OR KNN OR "gradient boosting" OR "Extreme Gradient Boost$" OR XGBoost OR AdaBoost OR "Light Gradient Boosting Machine" OR LightGBM OR "semi-supervised learning" OR "self-supervised learning" OR "representation learning" OR "contrastive learning" OR "unsupervised learning" OR kmeans OR k-means OR "hierarchical clustering" OR "agglomerative clustering" OR DBSCAN OR autoencoder$ OR "reinforcement learning" OR "few-shot learning" OR "transfer learning" OR "foundational models" OR "deep neural network" OR "convolutional neural network" OR CNN OR "recurrent neural network" OR "neural network analysis" OR RNN OR "long short-term memory" OR LSTM OR "attention mechanism" OR BERT OR GPT OR "graph neural network" OR GNN OR "ensemble learning" OR "multimodal learning" OR "ensemble model aggregation" OR "iterative model building" OR "categorical boosting$" OR CatBoost OR "Dimensionality Reduction" OR "Singular Value Decomposition" OR "Linear Discriminant Analys" OR (digital OR "data-driven") NEAR/3 (ph$enotyping))	Results: 2225396

#3 TS=((excessive OR increased OR daytime OR day-time OR diurnal ) NEAR/3 (sleepiness OR drowsiness OR somnolence ))	Results: 18730

#4 TS=("cataleptic attack*" OR cataplex* OR "disrupted nighttime sleep" OR "disrupted nocturnal sleep" OR Gelineau* OR Henneberg OR "insufficient nocturnal sleep" OR "insufficient sleep syndrome" OR "Kleine Levin" OR narcolep* OR neurolepsy OR "paroxysmal sleep" OR pseudonarcolep* OR "sleep inertia$" OR "sleeping beauty syndrome" OR "sleep epilepsy" )	Results: 10778

#5 TS=((hypersomn* OR hyper-somn* OR hypersleepiness ) NEAR/3 (idiopath* OR primary ))	Results: 810

#6 #3 OR #4 OR #5	Results: 27436

#7 #1 AND #2 AND #6	Results: 1305

----------------------------------------------------------------------

***************************

Database: Google Scholar

Search date: 05/06/2025

Search results: 200

Search Strategy:

----------------------------------------------------------------------

Keywords: (hypersomnia | "excessive somnolence" | narcolepsy | narcoleptic) ("machine learning" | "artificial intelligence" | "deep learning") (distinguish | diagnose | diagnosis | determine | determination | differentiate | recognize | classify | cluster | screen | detect | identify | phenotyping)

----------------------------------------------------------------------

## **Bibliography**

1.	Terzano MG, Parrino L, Sherieri A, et al. Atlas, rules, and recording techniques for the scoring of cyclic alternating pattern (CAP) in human sleep. *Sleep Medicine*. 2001;2(6):537-553. doi:10.1016/S1389-9457(01)00149-6

2.	Terzano MG, Mancia D, Salati MR, Costani G, Decembrino A, Parrino L. The Cyclic Alternating Pattern as a Physiologic Component of Normal NREM Sleep. *Sleep*. 1985;8(2):137-145. doi:10.1093/sleep/8.2.137

3.	Kales A, Rechtschaffen A, University of California LABIS, NINDB Neurological Information Network. *A Manual of Standardized Terminology, Techniques and Scoring System for Sleep Stages of Human Subjects. Allan Rechtschaffen and Anthony Kales, Editors*. U. S. National Institute of Neurological Diseases and Blindness, Neurological Information Network; 1968.

4.	Berry R, Brooks R, Gamaldo C, et al. The AASM Manual for the Scoring of Sleep and Associated Events: Rules, Terminology and Technical Specifications, Version 2.2. Published online 2015. www.aasmnet.org

5.	Gool JK, Zhang Z, Oei MSSL, et al. Data-Driven Phenotyping of Central Disorders of Hypersomnolence With Unsupervised Clustering. *Neurology*. 2022;98(23). doi:10.1212/WNL.0000000000200519

6.	Morand R, Fregolente L, Van Der Meer J, et al. iSPHYNCS: Unsupervised clustering in questionnaires and metadata reveals distinct subtypes in the narcolepsy borderland. *Neurology*. Preprint posted online March 25, 2025. doi:10.1101/2025.03.25.25324595

7.	Aellen FM, Van der Meer J, Dietmann A, Schmidt M, Bassetti CLA, Tzovara A. Disentangling the complex landscape of sleep–wake disorders with data-driven phenotyping: A study of the Bernese center. *European Journal of Neurology*. 2023;31(1):e16026. doi:10.1111/ene.16026

8.	Maruo T, Takagi S, Uchida S, Takahashi H, Sugihara G. Temporal patterns of sleep latency in central hypersomnia and attention deficit hyperactivity disorder: a cluster analysis exploration using Multiple Sleep Latency Test. *Front Psychiatry*. 2024;15:1361140. doi:10.3389/fpsyt.2024.1361140

9.	Liu D, Pang Z, Lloyd SR. A neural network method for detection of obstructive sleep apnea and narcolepsy based on pupil size and EEG. *IEEE TRANSACTIONS ON NEURAL NETWORKS*. 2008;19(2):308-318. doi:10.1109/TNN.2007.908634

10.	Šonka K, Šusta M, Billiard M. Narcolepsy with and without cataplexy, idiopathic hypersomnia with and without long sleep time: a cluster analysis. *Sleep Medicine*. 2015;16(2):225-231. doi:10.1016/j.sleep.2014.09.016

11.	Mengmeng W, Lanbo W, Weihan W, et al. Phenotypic clusters of narcolepsy type 1: Insights from age of onset, weight gain, sleep patterns, and impulsivity. *Sleep Medicine*. 2025;130:3-12. doi:10.1016/j.sleep.2025.02.047

12.	Cook JD, Rumble ME, Plante DT. Identifying subtypes of Hypersomnolence Disorder: a clustering analysis. *Sleep Medicine*. 2019;64:71-76. doi:10.1016/j.sleep.2019.06.015

13.	Baier PC, Sahlström H, Markström A, Furmark T, Bothelius K. Nocturnal sleep phenotypes in idiopathic hypersomnia – A data-driven cluster analysis. *Sleep Medicine*. 2024;124:127-133. doi:10.1016/j.sleep.2024.09.026

14.	Sharma M, Tiwari J, Patel V, Acharya UR. Automated Identification of Sleep Disorder Types Using Triplet Half-Band Filter and Ensemble Machine Learning Techniques with EEG Signals. *Electronics*. 2021;10(13):1531. doi:10.3390/electronics10131531

15.	Dimitriadis SI, Salis CI, Liparas D. An automatic sleep disorder detection based on EEG cross-frequency coupling and random forest model. *J Neural Eng*. 2021;18(4):046064. doi:10.1088/1741-2552/abf773

16.	Babu VS, Vaidya AS. Sleep Disorder Classification from EEG Signals using Fast Fourier Transform and Convolutional Neural Networks. In: *2024 2nd International Conference on Sustainable Computing and Smart Systems (ICSCSS)*. 2024:1472-1479. doi:10.1109/ICSCSS60660.2024.10625228

17.	Wadichar A, Murarka S, Shah D, et al. A Hierarchical Approach for the Diagnosis of Sleep Disorders Using Convolutional Recurrent Neural Network. *IEEE Access*. 2023;11:125244-125255. doi:10.1109/ACCESS.2023.3330901

18.	Newaz MdH, Rahman S, Bhuiyan MIH. A Machine Learning Approach for the Classification of Sleep Disorders Using Time and Frequency Domain Features of a Single Channel EEG Signal. In: *2025 International Conference on Electrical, Computer and Communication Engineering (ECCE)*. 2025:1-6. doi:10.1109/ECCE64574.2025.11013384

19.	Jain R, Ganesan RA. Effective diagnosis of sleep disorders using EEG and EOG signals. In: *2024 46th Annual International Conference of the IEEE Engineering in Medicine and Biology Society (EMBC)*. 2024:1-4. doi:10.1109/EMBC53108.2024.10782470

20.	Jain R, Ganesan RA. Effective Diagnosis of Various Sleep Disorders by LEE Classifier: LightGBM-EOG-EEG. *IEEE Journal of Biomedical and Health Informatics*. 2025;29(4):2581-2588. doi:10.1109/JBHI.2024.3524079

21.	Sharma M, Darji J, Thakrar M, Acharya UR. Automated identification of sleep disorders using wavelet-based features extracted from electrooculogram and electromyogram signals. *Computers in Biology and Medicine*. 2022;143:105224. doi:10.1016/j.compbiomed.2022.105224

22.	Albathan M. Sleep Disorder Diagnosis Through Complex-Morlet-Wavelet Representation Using Bi-GRU and Self-Attention. *ijacsa*. 2024;15(8). doi:10.14569/IJACSA.2024.0150814

23.	Cheng YH, Lech M, Wilkinson RH. Simultaneous Sleep Stage and Sleep Disorder Detection from Multimodal Sensors Using Deep Learning. *Sensors*. 2023;23(7):3468. doi:10.3390/s23073468

24.	Singh T, Jain R, Ganesan RA. Efficient Deep Learning Model for Classification of Patients with Sleep Disorders from Healthy Individuals. In: *IEEE 20th India Council International Conference (INDICON)*. 2023:329-333. doi:10.1109/INDICON59947.2023.10440817

25.	Wang W, Li J, Fang Y, Zheng Y, You F. A multimodal attention-fusion convolutional neural network for automatic detection of sleep disorders. *Appl Intell*. 2024;54(11-12):7086-7098. doi:10.1007/s10489-024-05499-7

26.	Olsen AV, Stephansen J, Leary E, et al. Diagnostic value of sleep stage dissociation as visualized on a 2-dimensional sleep state space in human narcolepsy. *Journal of Neuroscience Methods*. 2017;282:9-19. doi:10.1016/j.jneumeth.2017.02.004

27.	Christensen JAE, Munk EGS, Peppard PE, et al. The diagnostic value of power spectra analysis of the sleep electroencephalography in narcoleptic patients. *Sleep Medicine*. 2015;16(12):1516-1527. doi:10.1016/j.sleep.2015.09.005

28.	Hjuler Andersen L, Brink-Kjaer A, Sum-Ping O, et al. Probabilistic sleep staging in MSLTs across hypersomnia disorders. *Sleep*. 2025;48(2):zsae241. doi:10.1093/sleep/zsae241

29.	Liu Y, Zhang J, Lam V, et al. Altered Sleep Stage Transitions of REM Sleep: A Novel and Stable Biomarker of Narcolepsy. *Journal of Clinical Sleep Medicine*. 2015;11(08):885-894. doi:10.5664/jcsm.4940

30.	Chin WC, Huang SY, Liu FY, et al. The application of machine learning on brain imaging features of different narcolepsy subtypes. *Sleep*. 2024;47(2):zsad328. doi:10.1093/sleep/zsad328

31.	Fulong X, Chao L, Dianjiang Z, et al. Recursive Partitioning Analysis of Fractional Low-Frequency Fluctuations in Narcolepsy With Cataplexy. *Front Neurol*. 2018;9:936. doi:10.3389/fneur.2018.00936

32.	Gaultney JF, Gray ,David D., and Daley K. Improving predictive accuracy of a survey measure of risk for narcolepsy. *Health Psychology and Behavioral Medicine*. 2014;2(1):314-321. doi:10.1080/21642850.2014.892430

33.	Pan Y, Zhao D, Zhang X, et al. Machine learning-Based model for prediction of Narcolepsy Type 1 in Patients with Obstructive Sleep Apnea with Excessive Daytime Sleepiness. *NSS*. 2024;Volume 16:639-652. doi:10.2147/NSS.S456903

34.	Zhang Z, Mayer G, Dauvilliers Y, et al. Exploring the clinical features of narcolepsy type 1 versus narcolepsy type 2 from European Narcolepsy Network database with machine learning. *Sci Rep*. 2018;8(1):10628. doi:10.1038/s41598-018-28840-w

35.	Son DY, Kwon HB, Lee DS, et al. Changes in physiological network connectivity of body system in narcolepsy during REM sleep. *Computers in Biology and Medicine*. 2021;136:104762. doi:10.1016/j.compbiomed.2021.104762

36.	Coz AL, Vollhardt R, Champetier P, et al. EEG Signature of Idiopathic Hypersomnia: Insights from Sleep Microarchitecture and Hypnodensity Metrics. *bioRxiv*. Preprint posted online May 28, 2025:2025.05.26.656134. doi:10.1101/2025.05.26.656134

37.	Buettner R, Fuhrmann J, Kolb L. Towards high-performance differentiation between Narcolepsy and Idiopathic Hypersomnia in 10 minute EEG recordings using a Novel Machine Learning Approach. In: *2019 IEEE International Conference on E-Health Networking, Application & Services (HealthCom)*. 2019:1-7. doi:10.1109/HealthCom46333.2019.9009608

38.	Vilela M, Tracey B, Volfson D, et al. Identifying time‐resolved features of nocturnal sleep characteristics of narcolepsy using machine learning. *J Sleep Res*. 2024;33(6):e14216. doi:10.1111/jsr.14216

39.	Cesari M, Egger K, Stefani A, et al. Differentiation of central disorders of hypersomnolence with manual and artificial-intelligence-derived polysomnographic measures. *Sleep*. 2023;46(2):zsac288. doi:10.1093/sleep/zsac288

40.	Hédou J, Cederberg KL, Ambati A, et al. Proteomic biomarkers of Kleine–Levin syndrome. *Sleep*. 2022;45(9):zsac097. doi:10.1093/sleep/zsac097

41.	Schwartz AR, Cohen-Zion M, Pham LV, et al. Brief digital sleep questionnaire powered by machine learning prediction models identifies common sleep disorders. *Sleep Medicine*. 2020;71:66-76. doi:10.1016/j.sleep.2020.03.005

42.	Deng G, Niu M, Rao S, et al. A Unified Flexible Large Polysomnography Model for Sleep Staging and Mental Disorder Diagnosis. *medRxiv*. Preprint posted online May 8, 2025. doi:10.1101/2024.12.11.24318815

43.	Yu T, Gu Z, Huang R, et al. A Language Model Built on Sleep Stage Sequences Enables Efficient Sleep Assessment. *Psychiatry and Clinical Psychology*. Preprint posted online October 28, 2024. doi:10.1101/2024.10.26.24316166

44.	Stephansen JB, Olesen AN, Olsen M, et al. Neural network analysis of sleep stages enables efficient diagnosis of narcolepsy. *Nat Commun*. 2018;9(1):5229. doi:10.1038/s41467-018-07229-3

45.	Wang J, Zhao S, Zhou Y, et al. Narcolepsy Diagnosis With Sleep Stage Features Using PSG Recordings. *IEEE Transactions on Neural Systems and Rehabilitation Engineering*. 2023;31:3619-3629. doi:10.1109/TNSRE.2023.3312396
